# Synthesis and Mesomorphic Properties of Geometric and Conformation-Modulated Amphiphilic β-Cyclodextrin Liquid Crystals

**DOI:** 10.3390/molecules29235633

**Published:** 2024-11-28

**Authors:** Austin Che, Homayoun Ghaseminezhad, Carson O. Zellmann-Parrotta, Jessica Duong, Vance E. Williams, Chang-Chun Ling

**Affiliations:** 1 Department of Chemistry, University of Calgary, 2500 University Drive NW, Calgary, AB T2N 1N4, Canada; austin.che@ucalgary.ca (A.C.);; 2 Department of Chemistry, Simon Fraser University, Burnaby, BC V5A 1S6, Canada; homayoun_ghaseminezhad@sfu.ca (H.G.);

**Keywords:** amphiphilic cyclodextrin, liquid crystals, hexagonal columnar mesophase, cis/trans-alkenes

## Abstract

This study explores the liquid crystalline properties of novel amphiphilic β-cyclodextrin derivatives functionalized with seven oligoethylene glycol chains at the primary face, terminated with either an O-methyl or an O-cyanoethyl group, and fourteen hydrophobic aliphatic chains (elaidic or oleic acids) at the secondary face. These derivatives were designed to study the impact of chain conformation and terminal group polarity on their mesomorphic behavior. Thermal, microscopic, and X-ray diffraction studies revealed that the elaidic derivatives form columnar hexagonal mesophases, with the O-cyanoethyl derivative undergoing a slow, temperature-dependent transition to a bicontinuous cubic phase. The oleic derivatives, although less stable, also exhibit columnar hexagonal phases, but clear differences were observed in the clearing temperatures between these two groups of molecules, and they are also different from analogous derivatives containing no alkene functionalities. This work provides direct insights into the structure–mesomorphic property relationships of amphiphilic cyclodextrins in terms of the geometry and conformation flexibility of the hydrophobic regions, as well as the functional group attached to the end of the polar region.

## 1. Introduction

Cyclodextrins (CDs) are well recognized for their truncated cone-shaped hydrophobic cavities, which are sandwiched between two hydrophilic surfaces [1]. Remarkably, the narrower end of the hydrophilic surfaces of a CD (primary face) is formed with the more reactive primary hydroxyl groups of constituent D-glucopyranosyl building blocks, while the opposite end (wider, secondary face) is formed with the less reactive secondary hydroxyl groups (OH-2s and OH-3s) of the same building blocks. The regioselective modification of primary hydroxyl groups permits the introduction of different functional groups to opposite faces of the cavity in a programmable manner [2,3,4,5,6]; this structural feature differentiates CDs from most other macrocyclic hosts, making them unique and versatile scaffolds. A regioselective per-substitution either of the primary hydroxyl groups or of the secondary hydroxyl groups with the same functionalities would serve to pre-organize the functionalities, facilitating the creation of ordered materials. To take advantage of this, amphiphilic CD derivatives [7,8,9,10,11,12,13,14,15,16,17,18,19,20] have been developed as a class of very useful materials for drug delivery because of their ability to self-assemble in solutions into micelles, liposomes, and vesicles.

**Figure 1:**
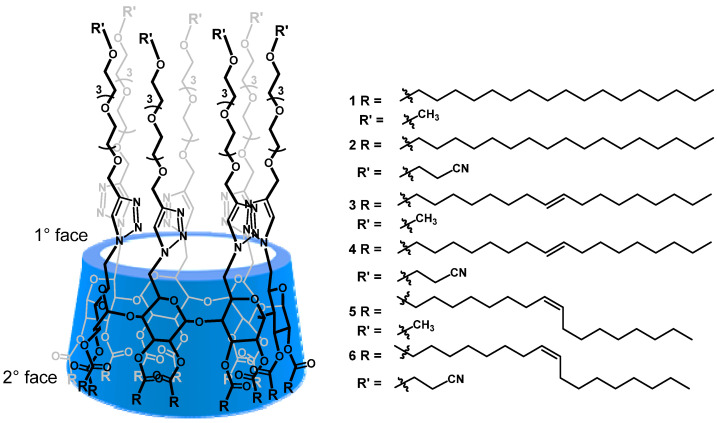
Structures of the target amphiphilic β-CD derivatives (**3**–**6**) bearing elaidic or oleic functionalities, with either a terminal methyl or a 2-cyanoethyl group on the tetraethylene glycol chain, in comparison with previously reported saturated analogs (**1**–**2**) [30].

In the solid state, amphiphilic CDs have been found to self-assemble into thermotropic liquid crystals (LCs) [21,22,23,24,25]. The first class of LCs based on amphiphilic CDs, reported in 1993 by Ling et al. [23], featured the covalent introduction of seven aliphatic chains to the primary face of β-CD via thioester linkages, forming a family of per-6-thioalkyl-substituted β-CDs bearing 14 secondary hydroxyl groups, and these amphiphilic CD materials, especially those bearing C18 and C16 alkyl lengths, showed a strong ability to self-assemble into smectic LCs above ~215 °C, but had very high clearing temperatures (>295 °C with decomposition). To elucidate the roles played by the hydrogen bond strength, a structure–LC property relationship study was subsequently carried out [26] by keeping the C18-thioalkyl substitution at the primary face, while substituting part or all of the secondary hydroxyl position alkyl groups of different sizes; they were all found to either completely lose their ability to self-assemble or to retain partially their ability to form LCs [26]. By shifting away from relying on inter/intramolecular H-bonding as the strong intermolecular forces to promote LC mesophases, during the last few years, we have started to pre-organize oligoethylene glycols (OEG) onto β-CD scaffolds using the well-known copper (I)-catalyzed Huisgen [2+3] dipolar cycloaddition reaction (“click” chemistry) [27,28], either linking them to the primary O-6 positions while leaving the other face per-functionalized with C18-aliphatic [29,30], or vice versa [31]. This has resulted in several families of amphiphilic CDs capable of forming LCs. It is worth noting that the mesophases formed by these materials greatly depend on the substitution patterns. For example, by placing seven C18-aliphatic chains at the primary face and fourteen OEG groups at the secondary face, we obtained amphiphilic CD derivatives capable of forming smectic mesophases, which showed enhanced fluidity and thermostability [31]. On the other hand, reversing the faces on which the polar OEG groups and apolar C18-aliphatic chains are attached dramatically changes the formed mesophases from smectic A (SmA) to columnar liquid crystals (Col_h_) [29,30]. Further chemical modifications have been carried out on the ends of OEG groups (such as compounds **1** and **2**, Figure 1) and the introduced chemical functionalities further influence the properties of the mesophases. These novel OEG-based materials have found different applications, such as proton or lithium conduction [30,32,33].

To further understand the structure–property relationship of amphiphilic CDs, in this study, we present the synthesis of a novel series of amphiphilic β-CDs that incorporate seven tetraethylene glycol groups at the primary face that are terminated with either a methyl or a 2-cyanoethyl group, and fourteen hydrophobic aliphatic chains embedded with a *trans*- or *cis*-alkene functionality (compounds **3**/**4**, elaidic acid and **5**/**6**, oleic acid, Figure 1). Compared to compounds containing only saturated aliphatic chains (compounds **1**/**2**, stearic acid, Figure 1), it is expected that the geometry and conformation flexibility of the unsaturated chains will be altered, which would further influence the packing/preorganization of these groups on a CD-scaffold, ultimately affecting the mesomorphic properties of the materials. Research reports showed that the incorporations of unsaturated *cis*-C_18_-aliphatic chains can influence the self-assembly behavior of glycosurfactants [34] and other LC materials [35,36,37]; additionally, it was reported that the incorporation of these functionalities could affect the drug loading in certain lipid nanoparticles [38].

## 2. Results and Discussion

### 2.1. Chemical Synthesis and Characterization

**Scheme 1:**
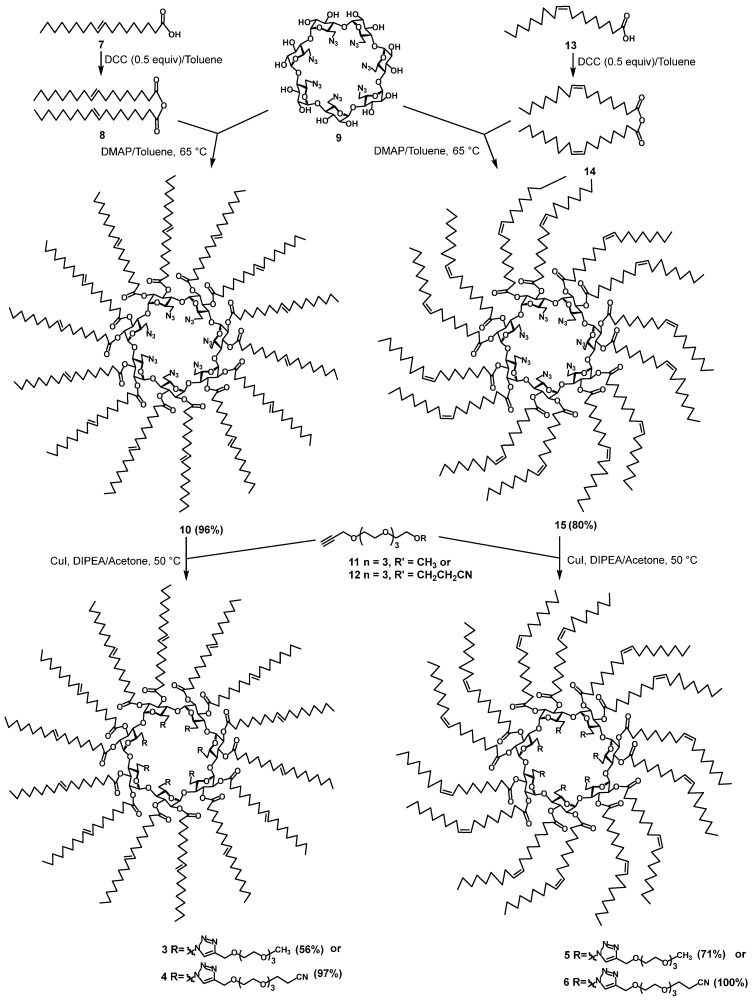
Synthetic scheme for β-CD targets **3**–**6** with 14 elaidate or 14 oleate ester groups on the secondary face and seven tetraethylene glycol chains, terminated with either a methyl or 2-cyanoethyl group, on the primary face.

The desired polyester targets **3**/**4** (per-2,3-*O*-elaidic esters) and **5**/**6** (per-2,3-*O*-oleyl esters) were synthesized according to Scheme 1. To introduce fourteen elaidate residues to the secondary face of per-6-azido-β-CD (**9**), the commercial elaidic acid (**7**) was activated to the corresponding anhydride (**8**) by a treatment with *N*,*N*′-dicyclohexylcarbodiimide as the dehydrating reagent in toluene at 65 °C. The crude anhydride (**8**, 2.0 equivalents per OH) was not purified, but combined with per-6-azido-β-CD (**8**) in the same solvent at 65 °C, using 4-*N*,*N*-dimethylpyridine as a base [39]. After 16 h, the resulting compound (**10**) was isolated with excellent yield (96%). The per-esterification of compound **10** was confirmed by ^1^H NMR (Figure S1), which showed a doublet at 5.08 ppm (*J* = 3.8 Hz) and two sets of doublets of doublets at 5.32 ppm (*J* = 9.4, 9.4 Hz) and 4.80 ppm (*J* = 10.1, 3.7 Hz), corresponding to the three types of protons, H-1s, H-3s, and H-2s, of the seven glucopyranosyl units of β-CD. Both signals that correspond to the seven H-2s and seven H-3s respectively, appear at a rather deshielded region, confirming the direct attachment of electron-withdrawing elaidate ester groups. A multiplet peak was observed at 5.45–5.37 ppm, corresponding to the unsaturated trans-double bond of the eladic ester chains. The fact that only one set of signal was observed for each type of protons is consistent with the presence of axial symmetry, confirming the per-O-esterifications at all O-2 and O-3 positions of the β-CD. Interestingly, in the C-13 NMR spectra (Figure S2), four peaks of alkenic carbons were observed, corresponding to the two types of double bonds embedded in elaidate groups that are attached to the O-2 and O-3 positions of the glucopyranosyl units.

The verification of complete per-2,3-*O*-estterifications in compound **10** was further conducted using mass spectroscopy. Unfortunately, high-resolution mass spectrometry using electrospray ionization (positive, ESI HRMS) failed to give us the peaks corresponding to the adducts of molecular ions, likely due to the high molecular weight and lack of adequate polar functionalities; however, low-resolution MALDI-TOF (positive) showed a peak at *m*/*z* 5031.3 (100%), which correlated closely with the anticipated sodium adduct of the expected structural formula C_294_H_511_N_21_O_42_Na (M + Na)^+^, which has a calculated *m*/*z* value of 5034.8 (100%), based on simulated isotope distributions of the expected compound **10**.

**Figure 2:**
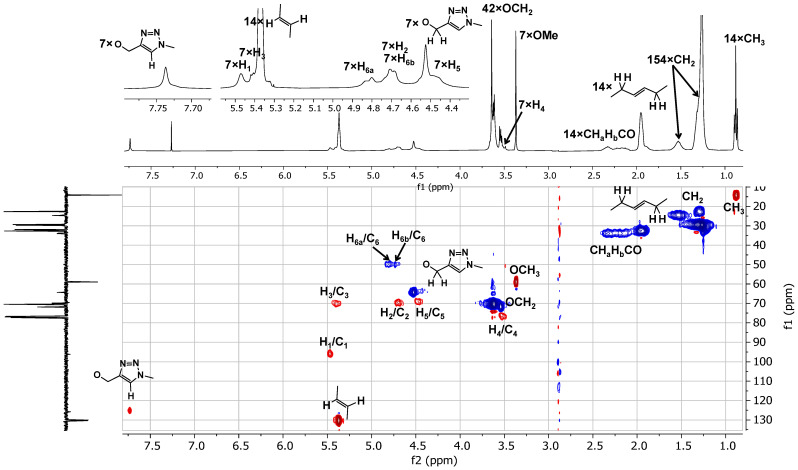
^1^H NMR Spectrum (**top**) and ^1^H-^13^HSQC spectrum (**bottom**) of compound **3**.

To obtain the targeted per-2,3-*O*-elaidate-modified compounds **3**/**4**, the mono-*O*-propargylated tetraethylene glycol derivatives **11** and **12** were synthesized as previously reported [30]. Each of these derivatives was then covalently linked to compound **10** via a copper (I)-catalyzed Huisgen [2+3] dipolar cycloaddition [27] in the presence of *N*,*N*-diisopropylethylamine (DIPEA) in acetone. The reaction was heated at 55 °C for approximately 96 h, and the desired amphiphilic β-CD derivatives (**3**–**4**) were successfully isolated. The copper catalyst was removed through multiple extractions with a 5% EDTA solution in water, followed by repeated precipitation–centrifugation in a mixture of dichloromethane and methanol. The final products were obtained in 56% and 97% yields. Structural characterizations of the final targets **3** and **4** were carried out using NMR experiments. As an example, Figure 2 shows the 1D ^1^H and 2D ^1^H-^13^C GHSQC correlation spectra of compound **3**.

As can be seen, the per-substitutions in compound **3** were supported by the presence of axial symmetry, as only one set of signals was observed for each type of proton in both ^1^H and ^13^C spectra. In the 1D ^1^H (Figure S5) and 2D ^1^H-^1^H GCOSY (Figure S7) spectra, the signals of the H-6a and H-6b protons of the glucopyranose units are observed at a highly deshielded region (~4.8 and ~4.7 ppm, respectively); this confirms the success of the [2+3] dipolar cycloaddition, as all the C-6s of the glucopyranose units are now directly attached to the newly formed aromatic 1,2,3-triazole rings, making them experience a deshielding effect due to the aromatic π-electrons. Similarly, the H-5 protons also experience some deshielding effect, as they are found to resonate at ~4.5 ppm. Most of the proton signals in the ^1^H-NMR spectra of compounds **3**–**4** exhibit broadness (Figures S5 and S9), attributed to the limitation of molecular rotation imposed by the substantial size of these molecules. Finally, the structures of compounds **3**–**4** were confirmed by high-resolution mass spectrometry (HRMS). For example, for compound **3**, HRMS (positive electrospray ionization, positive) showed a triple-charged peak at 2244.9593, which correlated well with the expected C_378_H_665_N_21_O_77_ (M + 3H)^3+^ molecular formula, which has a calculated *m*/*z* of 2244.9662 (M + 3H)^3+^. For compound **4**, the observed peak at *m*/*z* 2335.9890 also correlated well with the expected molecular formula C_392_H_672_N_28_O_77_ (M + 3H)^3+^, which has a calculated *m*/*z* at 2335.9916.

With the success of both elaidate-substituted targets **3** and **4**, we next continued our synthesis of the two oleate-substituted targets **5** and **6**. As shown in Scheme 1, the desired oleic anhydride **14** was first prepared from the commercially available oleic acid (**13**), as above. The crude anhydride (2.0 equiv. per OH) was then directly used to carry out polyesterification of all O-2 and O-3 positions of per-6-azido-β-CD (**9**) using the same conditions to afford the key intermediate **15**, which was isolated in pure form (80% yield). The structure of compound **15** was characterized similarly, using NMR spectroscopy (See Figures S13–S16) and low-resolution MALDI-TOF mass spectrometry, which showed the anticipated sodium adduct peak at *m*/*z* 5029.6 (100%), corresponding closely to the expected structure of compound **15**, which has the molecular formula C_294_H_511_N_21_O_42_Na (M + Na)^+^, with a calculated *m*/*z* of 5034.8 (100%).

With the key compound **15** in hand, we subjected it to reactions with the two mono-*O*-propargylated tetraethylene glycol derivatives **11** and **12**, using the copper (I)-catalyzed Huisgen [2+3] dipolar cycloaddition in a similar manner as above. After 96 h, the targeted amphiphilic β-CD derivatives (**5**–**6**) were successfully formed and isolated in 71% and almost quantitative yields, respectively. Structural characterizations of the final targets **5** and **6** were also carried out using NMR experiments (see experiments, Figures S17–S24) and HRMS. Notably, the alkene protons on the *cis*-double bonds were observed at the 5.20–5.41 ppm regions (Figures S17 and S21) and HRMS (positive electrospray ionization, positive) also confirmed the structural identification of both amphiphilic targets: with compound **5** having a peak observed at *m*/*z* 2244.9490 (calculated *m*/*z* for C_378_H_665_N_21_O_77_ (M + 3H)^3+^ ion: 2244.9662) and compound **6** having a peak observed at *m*/*z* 2335.9915 (calculated *m*/*z* for C_392_H_672_N_28_O_77_ (M + 3H)^3+^ ion: 2335.9782).

### 2.2. Mesomorphic Properties

**Figure 3:**
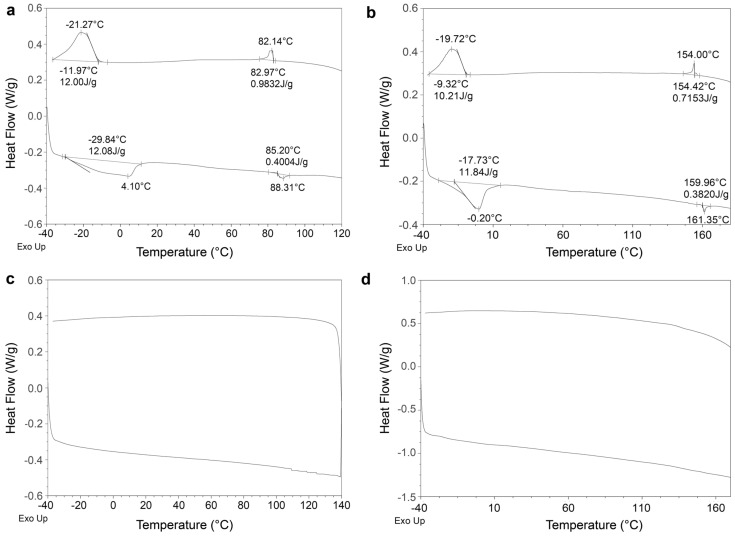
DSC thermograms of (**a**) **3**, (**b**) **4**, (**c**) **5**, and (**d**) **6**.

**Figure 4:**
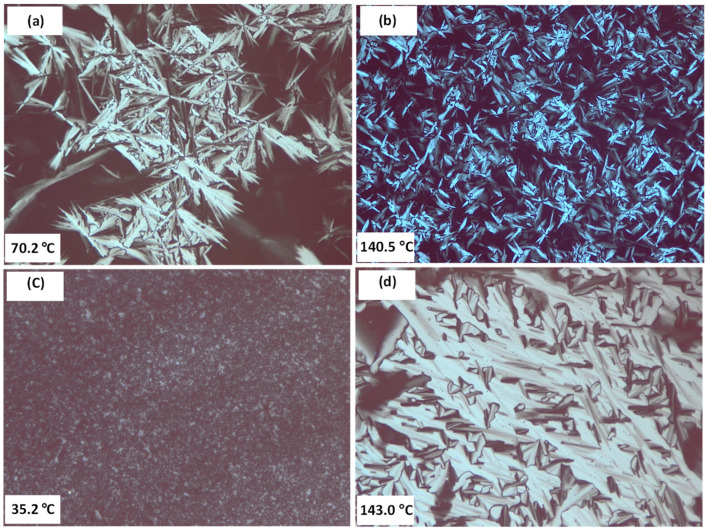
Polarized micrographs showing the cooling cycles of (**a**) compound **3** at 70.2 °C, (**b**) compound **4** at 140.5 °C, (**c**) compound **5** at 35.2 °C, and (**d**) compound **6** at 143.0 °C, with all compounds in the Col_h_ phase.

**Figure 5:**
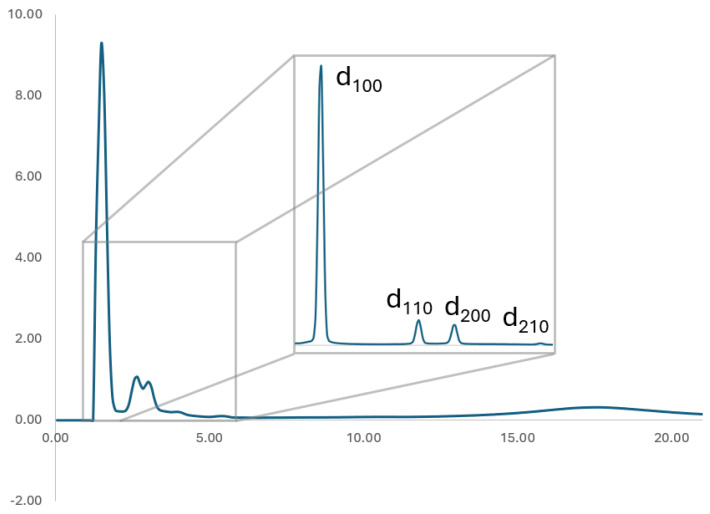
XRD of compound **3** at room temperature.

The DSC thermogram of compound **3** (Figure 3a) displayed two endothermic transitions upon heating: a broad peak at ~4 °C and a second, lower-intensity peak at 88.3 °C. Integrations of the two peaks revealed a large enthalpic change (81.3 kJ/mol) for the lower transition and a much smaller enthalpic change (2.7 kJ/mol) for the higher transition (Table 1). Polarized optical microscopy (POM) revealed that the latter transition corresponded to the change from a birefringent liquid crystal phase to an isotropic liquid. POM images of the liquid crystal phase of compound **3** during the cooling cycle exhibited fan-shaped domains, characteristic of a columnar hexagonal (Col_h_) phase (Figure 4a and Figure S29). This phase assignment was confirmed by variable temperature X-ray diffraction (vt-XRD), which showed four distinct peaks in the low-angle region that indexed to the (100), (110), (200), and (210) reflections of a Col_h_ liquid crystal (Figure 5 and Table 2). Due to instrumental limitations, we were unable to investigate the lower-temperature phase, denoted as **X** (Table 1), by vt-XRD or POM.

Compound **4** exhibits more complex phase behavior. Upon heating, compound **4** exhibits two transitions in the DSC (Figure 3b), at −0.2 °C (79.7 kJ/mol) and 161.4 °C (2.6 kJ/mol); the latter transition was identified as the clearing temperature to the isotropic phase, which was confirmed by POM and XRD experiments. Upon cooling from the isotropic phase, the POM images show fluid fan-shaped textures typical of a columnar hexagonal (Col_h_) phase (Figure 4b and Figure S29). The X-ray diffractogram of this liquid crystal shows three low-angle peaks, indexed to the (100), (110), and (200) Miller planes of a Col_h_ structure (Table 2). Both the POM textures and XRD patterns initially remain unchanged upon cooling to room temperature. However, samples left at room temperature for extended periods (days to weeks) gradually developed additional diffraction peaks, which index to a bicontinuous cubic phase (Cub_bi_, *1a3d* space group) [40,41,42,43]. This is likely because the high viscosity of the Col_h_ phase slows the transition to the Cub_bi_ phase during cooling. As evidence, room-temperature POM images show a progressive decrease in birefringence (Figure 4 and Figure S29), consistent with a slow transformation from the Col_h_ phase to an optically isotropic and more thermally stable Cub_bi_ phase. Upon reheating, the Cub_bi_ phase remains optically isotropic until ~65 °C, where a sudden increase in birefringence is observed (Figure 4 and Figure S29), and the POM textures gradually increase in brightness upon further heating (Figure 4 and Figure S29), indicating the reformation of the columnar phase, although no corresponding transition was detected by DSC. Finally, compound **4** completely clears into isotropic liquid phase at 160.3 °C (Figure 4 and Figure S29). This phase behavior, including the supercooling of the columnar phase, is reproducible across subsequent heating and cooling cycles. Similarly to compound **3**, the transition at −0.2 °C to an unknown phase, which is denoted as **X** (Table 1), could not be directly examined by POM or XRD due to instrumental limitations.

No phase transitions were observed in the DSC traces for compounds **5** and **6** (Figure 3c,d), which is likely to have been due to the very small enthalpy changes between the relatively disordered Col_h_ phase and the isotropic liquid. The melting transitions of compounds **5** and **6** could be below the detectable range of our DSC experiments, which is further supported by the absence of crystallization observations. However, the POM studies revealed the characteristic textures of columnar hexagonal (Col_h_) phases for both compounds (Figure 4c for compound **5**, Figure 4d for compound **6**; also see Figure S29), with compound **5** having a clearing transition at 51 °C, and at 150 °C for compound **6**.

The XRD pattern for **5** showed two low-angle peaks, indexed to the (100) and (110) reflections of a Col_h_ phase (Table 2). These peaks were significantly broadened, indicating a relatively disordered sample, which is likely to contribute to the compound’s tendency to polymerize. However, it should be noted that no significant polymerization occurred during the POM experiment for compound **5**, as the POM experiments can be repeated and the sample remained birefringent and fluid during cooling cycle. The polymerization of compound **5** was confirmed by dissolving a small amount of the nearly insoluble material and analyzing the ^1^H NMR spectra. The spectra displayed asymmetrical peaks and a reduction in the integration values of the cis-alkene protons, further supporting polymerization. In contrast, the XRD pattern of compound **6** exhibited three sharp peaks corresponding to the (100), (110), and (200) reflections of a well-ordered Col_h_ phase (Table 2). Compound **6** was also observed undergoing polymerization, but this occurred over a much longer period (several months). No polymerization was observed during the POM and XRD experiments for compound **6**.

The clearing temperatures of each compound are also influenced by the geometry of the alkene functionalities of the aliphatic chains region and the terminal group of the oligoethylene glycol segments. Using the two *O*-methyl-terminated derivatives as an example, the compound containing the trans-alkene functionalities, **3**, has a higher clearing temperature (88.3 °C) than its cis-alkene analog **5**, which clears at 51 °C, but lower than the 107.7 °C (previously reported for the *O*-methylated analog **1** [30]), which contains only fully saturated octadecanoyl groups. The same trend was observed for the three *O*-2-cyanoethyl-terminated derivatives (**4** (trans) clears at 161.4 °C, **6** (cis) clears at 150 °C, and fully saturated compound **2** clears at 167.3 °C [30]). These results indicate that, compared to the amphiphilic CD derivatives containing cis-alkene functionalities, the analogs containing the linear trans-alkene chains have stronger van der Waals forces, because higher orders can be established among the trans-alkene-functionalized aliphatic chains to allow better packing efficiency, whereas higher degrees of disorder take place among the cis-alkene-functionalized analogs due to their bent geometry. On the other hand, the two compounds containing fully saturated *n*-octadecyl chains consistently exhibit the highest clearing temperatures in each family, suggesting that the higher conformation flexibility of the fully saturated *n*-octadecyl chains permits more efficient packing. Finally, all three amphiphilic CD derivatives containing *O*-2-cyanoethyl-terminated tetraethylene glycol groups exhibit significantly higher clearing temperatures than their *O*-methyl derivatives; this difference is ascribed to the much larger dipole moment of the nitrile groups compared to the methyl groups.

## 3. Conclusions

In this study, we successfully synthesized a series of novel amphiphilic β-CD derivatives featuring seven O-methyl- or O-2-cyanoethyl-functionalized tetraethylene glycol groups at the primary face and fourteen elaidic or oleic chains at the secondary face of β-CD. The availability of these series of amphiphilic CD derivatives enabled a first-hand appreciation of their liquid crystalline properties. Our results demonstrate that the phase behavior of these materials is strongly influenced by the geometry of the aliphatic chains and the terminal group of the OEG segments. The elaidic derivatives exhibit stable columnar hexagonal phases, with compound **4** undergoing a transformation to a bicontinuous cubic phase over time, highlighting its unique phase transition dynamics. The oleic derivatives also show columnar mesophases but display lower stability and a tendency to polymerize (especially compound **5**). The presence of nitrile groups significantly increases the clearing temperatures of these materials due to the larger dipole moment. These findings contribute to the broader understanding of the role of chain geometry/conformation flexibility and terminal group polarity in determining the mesomorphic properties of amphiphilic CDs and open new possibilities for their application in advanced materials design.

## 4. Materials and Methods

### 4.1. Methods

**General methods.** All commercial reagents were used as supplied unless otherwise stated. Analytical thin-layer chromatography was performed on Silica Gel 60-F_254_ (Sigma-Aldrich^®^ TLC Plates, Oakville, ON, Canada) with detection by quenching of fluorescence and/or by charring with 5% sulfuric acid in water or with a ceric ammonium molybdate dip. Column chromatography was performed on Silica Gel 60 (Silicycle, Quebec City, QC, Canada). Organic solutions from extractions were concentrated under vacuum with the assistance of a heat bath. The ^1^H NMR spectra were recorded at 400 MHz and ^13^C NMR spectra were recorded at 100 MHz on a Bruker spectrometer. Chemical shifts δ_H_ and δ_C_ are reported in δ (ppm) and referenced to residual CHCl_3_ (δ_H_ 7.24, δ_C_ 77.0, CDCl_3_). First-order coupling constants were reported in Hz for proton nuclei. The ^1^H and ^13^C NMR spectra were assigned with the assistance of DEPTQ, COSY, and HSQC spectra. Low-resolution mass spectra were obtained using mass spectrometry with Autoflex III Smartbeam (Bruker Daltonics Inc., Billerica, MA, USA) matrix-assisted laser desorption/ionization (MALDI-TOF/TOF). High-resolution ESI-QTOF mass spectra were recorded on an Agilent 6520 Accurate Mass Quadrupole Time-of-Flight LC/MS spectrometer.

**DSC**. Phase transition temperatures and enthalpies were investigated using differential scanning calorimetry (DSC) on a DSC Q200 instrument (TA Instruments, New Castle, DE, USA) equipped with a refrigerated cooling system (TA Instruments, Refrigerated Cooling System 90). Heating and cooling measurements were performed at a rate of 10 °C/min. DSC thermograms of the first heating and cooling cycles for all compounds are shown in the main text, while any subsequent runs are shown in the ESI.

**POM**. Polarized optical microscopy experiments were carried out using an Olympus BX50 microscope equipped with a Nikon D90 DSLR camera. Sample temperatures were controlled using a Linkam LTS350 heating stage coupled with a TMS94 temperature controller.

**XRD**. XRD measurements were conducted on a SAXSLAB Ganesha 300XL small-angle X-ray scattering (SAXS) instrument (Cu source, 45 kV, 0.6 mA). All samples were loaded into thin-walled quartz capillary tubes (Charles Supper Company, Westborough, MA, USA) with an outer diameter of 1.5 mm. All measurements were performed on a Linkam T95-PE heating stage. Each spectrum was collected for 8 min.

### 4.2. Chemical Synthesis

**Compound 10.**

Elaidic acid (**7**, 3.02 g, 10.69 mmol) was dissolved in anhydrous toluene (40 mL). DCC (1.10 g, 5.35 mmol) was added to the reaction flask and the reaction was stirred for 4 h under an inert argon atmosphere at 65 °C. The solid precipitate was filtered off and the filtrate containing anhydride **8** was reheated to 65 °C. Compound **9** (0.25 g, 0.19 mmol) and DMAP (1.47 g, 12.02 mmol) were added to the warm solution and the reaction was stirred overnight under an inert argon atmosphere. The reaction flask was concentrated before adding methanol (3.0 mL) to form a precipitate. The solution was centrifuged and the solid was redissolved in DCM (5 mL) and precipitated again with methanol (30 mL). The precipitate was precipitated and centrifuged twice more to afford compound **10** as a solid (0.92 g, 96%). R_f_ = 0.58 (EtOAc:Hexanes, 10:90). [α]_25_^D^ + 54.4 (*c* 0.57, CHCl_3_). ^1^H NMR (400 MHz, CDCl_3_) *δ* 5.45–5.37 (m, 28H, 28 × C=CH), 5.32 (dd, *J* = 9.4, 9.4 Hz, 7H, 7 × H-3), 5.08 (d, *J* = 3.8 Hz, 7H, 7 × H-1), 4.80 (dd, *J* = 10.1, 3.7 Hz, 7H, 7 × H-2), 4.03 (br ddd, 7H, 7 × H-5), 3.80–3.69 (m, 14H, 7 × H-4, 7 × H-6_a_), 3.64 (br dd, 7H, 7 × H-6_b_), 2.44–2.11 (m, 28H, 14 × CH_2_COO), 2.03–1.93 (m, 56H, 28 × C=CHC*H*_2_), 1.65–1.52 (m, 28H, 14 × C*H*_2_CH_2_COO), 1.42–1.22 (m, 280H, 140 × CH_2_), 0.90 (t, *J* = 7.1 Hz, 42H, 14 × CH_3_). ^13^C NMR (101 MHz, CDCl_3_) *δ* 173.4, 171.9, 130.48, 130.46, 130.10, 130.07, 96.5, 76.8, 70.9, 70.4, 70.3, 51.7, 34.3, 34.0, 32.8, 32.1, 29.96, 29.94, 29.87, 29.7, 29.60, 29.57, 29.50, 29.44, 29.40, 29.37, 25.0, 24.9, 22.9, 14.3. LRMS (MALDI-TOF, positive) *m*/*z* calc’d for C_294_H_511_N_21_O_42_Na (M + Na^+^): 5031.8 (15.65%) and 5034.849 (100%); found 5031.3.

**Compound 3**

Compound **11** [30] (0.14 g, 0.56 mmol), DIPEA (4.9 µL, 27.95 µmol), and CuI (5.3 mg, 27.95 µmol) were added to a solution of compound **10** (0.20 g, 39.93 µmol) dissolved in acetone (7.0 mL). The reaction was heated to 55 °C and stirred for 4 d. The solution was concentrated and then diluted with dichloromethane (75 mL) and washed with 5% ethylenediamine-*N*,*N*,*N*′,*N*′-tetraacetic acid in water (2 × 75 mL). The organic phase was dried with sodium sulfate before being evaporated and then redissolved in DCM (2 mL), before the addition of methanol (30 mL) to form a precipitate, which was centrifuged and decanted. Precipitation and centrifugation were repeated two more times to obtain the target compound 3 as a waxy solid (0.15 g, 56%). R_f_ = 0.63 (CH_3_OH:CH_2_Cl_2_, 10:90). [α]_25_^D^ + 19.2 (*c* 0.50, CHCl_3_). ^1^H NMR (400 MHz, CDCl_3_) δ 7.74 (s, 7H, 7 × 1,2,3-triazole), 5.52–5.27 (m, 42H, 7 × H-1, 7 × H-3, 28 × C=CH), 4.89–4.61 (m, 21H, 7 × H-2, 7 × H-6_a_, 7 × H-6_b_), 4.58–4.40 (m, 21H, 7 × H-5, 7 × OCH_2_-1,2,3-triazole), 3.71–3.43 (m, 119H, 7 × H-4, 56 × OCH_2_), 3.37 (s, 21H, 7 × OCH_3_), 2.43–2.06 (m, 28H, 14 × CH_2_COO), 2.01–1.82 (m, 56H, 28 × C=CHC*H*_2_), 1.63–1.42 (m, 28H, 14 × C*H*_2_CH_2_COO), 1.39–1.16 (m, 280H, 140 × CH_2_), 0.88 (t, *J* = 7.0 Hz, 42H, 14 × CH_3_). ^13^C NMR (101 MHz, CDCl_3_) δ 173.0, 171.7, 130.41, 130.37, 130.08, 130.02, 125.8, 96.2, 71.9, 70.53, 70.48, 70.45, 70.37, 69.8, 69.5, 64.4, 59.0, 34.0, 33.9, 32.71, 32.66, 31.9, 29.8, 29.7, 29.52, 29.46, 29.43, 29.39, 29.33, 29.29, 29.24, 29.22, 24.7, 24.6, 22.7, 14.1. HRMS (ESI-QTOF, positive) *m*/*z* calc’d for C_378_H_665_N_21_O_77_ (M + 3H^+^): 2244.9662 found 2244.9593.

**Compound 4**

Compound **12** [30] (0.14 g, 0.56 mmol), DIPEA (4.9 µL, 27.95 µmol), and CuI (5.3 mg, 27.95 µmol) were added to a solution of compound **10** (0.20 g, 39.93 µmol) dissolved in acetone (6.0 mL). The reaction was heated to 55 °C and stirred for 4 d. The solution was concentrated and then diluted with dichloromethane (40 mL) and washed with 5% ethylenediamine-*N*,*N*,*N*′,*N*′-tetraacetic acid in water (2 × 100 mL). The aqueous phased was washed with DCM (25 mL) and separated. The organic extracts were combined and dried with sodium sulfate before being evaporated. The crude product was redissolved in DCM (2 mL) before the addition of methanol (35 mL) to form a precipitate, which was centrifuged and decanted. Precipitation and centrifugation were repeated two more times to obtain the target compound 4 as a solid (0.27 g, 97%). R_f_ = 0.44 (CH_3_OH:CH_2_Cl_2_, 10:90). [α]_25_^D^ + 22.2 (*c* 0.40, CHCl_3_). ^1^H NMR (400 MHz, CDCl_3_) δ 7.73 (s, 7H, 7 × 1,2,3-triazole), 5.50–5.26 (m, 42H, 7 × H-1, 7 × H-3, 28 × C=CH), 4.86–4.61 (m, 21H, 7 × H-2, 7 × H-6_a_, 7 × H-6_b_), 4.56–4.40 (m, 21H, 7 × H-5, 7 × OCH_2_-1,2,3-triazole), 3.75–3.44 (m, 133H, 7 × H-4, 63 × OCH_2_), 2.61 (t, *J* = 6.3 Hz, 14H, 7 × CH_2_CN), 2.42–2.01 (m, 28H, 14 × CH_2_COO), 1.99–1.87 (m, 56H, 28 × C=CHC*H*_2_), 1.61–1.42 (m, 28H, 14 × C*H*_2_CH_2_COO), 1.37–1.17 (m, 280H, 140 × CH_2_), 0.86 (t, *J* = 7.1 Hz, 42H, 14 × CH_3_). ^13^C NMR (101 MHz, CDCl_3_) δ 173.0, 171.7, 144.6, 130.41, 130.36, 130.06, 130.00, 125.8, 118.0, 96.2, 70.7, 70.6, 70.47, 70.45, 70.35, 65.9, 64.3, 34.0, 33.8, 32.70, 32.68, 32.65, 31.9, 29.8, 29.7, 29.50, 29.45, 29.41, 29.38, 29.32, 29.27, 29.23, 29.21, 24.7, 24.6, 22.7, 18.8, 14.1. HRMS (ESI-QTOF, positive) *m*/*z* calc’d for C_392_H_672_N_28_O_77_ (M + 3H^+^): 2335.9916; found 2335.9890.

**Compound 15**

Oleic acid (**13**, 12.08 g, 42.77 mmol) was dissolved in anhydrous toluene (200 mL). DCC (4.41 g, 21.38 mmol) was added to the reaction flask and the reaction was stirred for 3.5 h under an inert argon atmosphere at 65 °C. The solid precipitate was filtered off and the filtrate containing the intermediate anhydride **14** was reheated to 65 °C. Compound **9** (1.0 g, 0.76 mmol) and DMAP (5.22 g, 42.77 mmol) was added to the warm solution and the reaction was stirred for 24 h under an inert argon atmosphere. The reaction flask was concentrated before adding methanol (200 mL) to form a precipitate. The solution was decanted, redissolved in DCM (5 mL) and precipitated again with methanol (100 mL). The precipitate was precipitated and centrifuged twice more to afford compound **15** as a solid (3.06 g, 80%). R_f_ = 0.40 (EtOAc:Hexanes, 10:90). [α]_25_^D^ + 68.4 (*c* 0.58, CHCl_3_). ^1^H NMR (400 MHz, CDCl_3_) δ 5.42–5.26 (m, 35H, 7 × H-3, 28 × C=CH), 5.07 (d, *J* = 3.8 Hz, 7H, 7 × H-1), 4.79 (dd, *J* = 10.1, 3.7 Hz, 7H, 7 × H-2), 4.00 (br ddd, 7H, 7 × H-5), 3.79–3.67 (m, 14H, 7 × H-4, 7 × H-6_a_), 3.63 (br dd, 7H, 7 × H-6_b_), 2.42–2.12 (m, 28H, 14 × CH_2_COO), 2.01 (m, 56H, 28 × C=CHC*H*_2_), 1.64–1.49 (m, 28H, 14 × C*H*_2_CH_2_COO), 1.40–1.20 (m, 280H, 140 × CH_2_), 0.93–0.83 (t, *J* = 7.2 Hz, 42H, 14 × CH_3_). ^13^C NMR (101 MHz, CDCl_3_) δ 173.1, 171.7, 129.98, 129.95, 129.57, 129.53, 96.3, 76.6, 70.7, 70.3, 70.1, 51.6, 34.1, 33.8, 31.9, 29.87, 29.84, 29.8, 29.6, 29.5, 29.4, 29.34, 29.32, 29.27, 29.2, 27.3, 27.2, 24.8, 24.7, 22.7, 14.1. LRMS (MALDI-TOF, positive) *m*/*z* calc’d for C_294_H_511_N_21_O_42_Na (M + Na^+^): 5031.8 (15.65%) and 5034.849 (100%); found 5029.6.

**Compound 5**

Compound **11** (0.14 g, 0.56 mmol), DIPEA (4.9 µL, 27.95 µmol), and CuI (5.3 mg, 27.95 µmol) were added to a solution of compound **15** (0.20 g, 39.93 µmol) dissolved in acetone (5.0 mL). The reaction was heated to 55 °C and stirred for 4 d. The solution was concentrated and then diluted with ethyl acetate (100 mL) and washed with 5% ethylenediamine-*N*,*N*,*N*′,*N*′-tetraacetic acid in water (3 × 100 mL). The organic phase was dried with sodium sulfate before being evaporated and then redissolved in DCM (2 mL) before the addition of methanol (35 mL) to form a precipitate, which was centrifuged and decanted. Precipitation and centrifugation were repeated two more times to obtain the target compound **5** as a waxy solid (0.19 g, 71%). R_f_ = 0.63 (CH_3_OH:CH_2_Cl_2_, 10:90). [α]_25_^D^ + 17.0 (*c* 0.44, CHCl_3_). ^1^H NMR (400 MHz, CDCl_3_) δ 7.72 (s, 7H, 7 × 1,2,3-triazole), 5.47 (br d, 7H, 7 × H-1), 5.41–5.24 (m, 35H, 7 × H-3, 28 × C=CH), 4.87–4.62 (m, 21H, 7 × H-2, 7 × H-6_a_, 7 × H-6_b_), 4.58–4.37 (m, 21H, 7 × H-5, 7 × OCH_2_-1,2,3-triazole), 3.69–3.46 (m, 119H, 7 × H-4, 56 × OCH_2_), 3.36 (s, 21H, 7 × OCH_3_), 2.42–2.06 (m, 28H, 14 × CH_2_COO), 1.99 (m, 56H, 28 × C=CHC*H*_2_), 1.64–1.43 (m, 28H, 14 × C*H*_2_CH_2_COO), 1.38–1.17 (m, 280H, 140 × CH_2_), 0.92–0.81 (t, *J* = 7.1 Hz, 42H, 14 × CH_3_). ^13^C NMR (101 MHz, CDCl_3_) δ 173.0, 171.6, 144.7, 129.93, 129.89, 129.57, 129.51, 125.7, 96.2, 76.7, 71.9, 70.5, 70.48, 70.45, 70.37, 69.8, 64.4, 59.0, 34.0, 33.7, 31.9, 29.9, 29.8, 29.54, 29.52, 29.45, 29.40, 29.33, 29.30, 29.27, 29.2, 27.3, 27.24, 27.21, 24.8, 24.6, 22.7, 14.1. HRMS (ESI-QTOF, positive) *m*/*z* calc’d for C_378_H_665_N_21_O_77_ (M + 3H^+^): 2244.9662; found 2244.9490.

**Compound 6**

Compound **12** (0.14 g, 0.56 mmol), DIPEA (4.9 µL, 27.95 µmol), and CuI (5.3 mg, 27.95 µmol) were added to a solution of compound **15** (0.20 g, 39.93 µmol) dissolved in acetone (6.0 mL). The reaction was heated to 55 °C and stirred for 4 d. The solution was concentrated and then diluted with dichloromethane (40 mL) and washed with 5% ethylenediamine-*N*,*N*,*N*′,*N*′-tetraacetic acid in water (2 × 100 mL). The organic phase was dried with sodium sulfate before being evaporated and then redissolved in DCM (2 mL) before the addition of methanol (35 mL) to form a precipitate, which was centrifuged and decanted. Precipitation and centrifugation were repeated two more times to obtain the target compound **6** as a waxy solid (0.29 g, 100%). R_f_ = 0.47 (CH_3_OH:CH_2_Cl_2_, 10:90). [α]_25_^D^ + 20.1 (*c* 0.61, CHCl_3_). ^1^H NMR (400 MHz, CDCl_3_) δ 7.75 (s, 7H, 7 × 1,2,3-triazole), 5.53–5.20 (m, 42H, 7 × H-1, 7 × H-3, 28 × C=CH), 4.85–4.58 (m, 21H, 7 × H-2, 7 × H-6_a_, 7 × H-6_b_), 4.57–4.39 (m, 21H, 7 × H-5, 7 × OCH_2_-1,2,3-triazole), 3.75–3.45 (m, 133H, 7 × H-4, 63 × OCH_2_), 2.60 (t, *J* = 6.4 Hz, 14H, 7 × CH_2_CN), 2.39–2.04 (m, 28H, 14 × CH_2_COO), 2.05–1.87 (m, 56H, 28 × C=CHC*H*_2_), 1.63–1.40 (m, 28H, 14 × C*H*_2_CH_2_COO), 1.37–1.12 (m, 280H, 140 × CH_2_), 0.85 (t, *J* = 6.7 Hz, 42H, 14 × CH_3_). ^13^C NMR (101 MHz, CDCl_3_) δ 173.0, 171.7, 144.5, 129.94, 129.90, 129.55, 129.48, 125.9, 118.0, 96.2, 76.8, 70.62, 70.55, 70.4, 70.3, 69.8, 69.5, 65.9, 64.3, 50.6, 50.0, 34.0, 33.7, 31.9, 29.87, 29.85, 29.7, 29.53, 29.50, 29.44, 29.39, 29.32, 29.29, 29.2, 27.3, 27.2, 24.8, 24.6, 22.6, 18.8, 14.1. HRMS (ESI-QTOF, positive) *m*/*z* calc’d for C_392_H_672_N_28_O_77_ (M + 3H^+^): 2335.9916; found 2335.9782.

## Figures and Tables

**Table 1:** Phase behavior of compounds **3**–**6**. Unless otherwise noted, transition temperatures and enthalpies (in parentheses) were determined by DSC on the first heating run at a scan rate of 10 °C/min.

Compound		Phase Transitions
**3**	Heating	**X** ~4 °C (81.3 kJ/mol) **Col_h_** 88.3 °C (2.7 kJ/mol) **Iso**
	Cooling	**Iso** 82.1 °C (6.6 kJ/mol) **Col_h_** −21.3 °C (86.8 kJ/mol) **X**
**4**	Heating	**X** −0.2 °C (79.7 kJ/mol) **Cub_bi_** ~70 °C ^a^ **Col_h_** 161.4 °C (2.6 kJ/mol) **Iso**
	Cooling	**Iso** 154.0 °C (4.8 kJ/mol) **Col_h_** −19.7 °C (68.7 kJ/mol) **X**
**5**	Heating	**Col_h_** 51 °C ^a^ **Iso**
	Cooling	**Iso** 44 °C ^a^ **Col_h_**
**6**	Heating	**Col_h_** 150 °C ^a^ **Iso**
	Cooling	**Iso** 147 °C ^a^ **Col_h_**

^a^ Determined by POM. **Col_h_**: hexagonal columnar mesophase; **Cub_bi_**: bicontinuous cubic mesophase; **X**: an unknown phase; **Iso**: isotropic phase.

**Table 2:** Summary of X-ray diffraction data for compounds **3**, **4**, **5**, and **6**, showing d-spacing values, Miller indices, and the identified phases at various temperatures. The table includes lattice constants for each compound phase where applicable.

	T (°C)	d-Spacing (Å)	Miller Index (hkl)	Phase (Lattice Constants)
**3**	70	49.57	(100)	Col_h_ (a = 57.2 Å)
		28.55	(110)	
		24.64	(200)	
		18.61	(210)	
		5.1	alkyl halo	
**4**	140	52.5	(100)	Col_h_ (a = 60.6 Å)
		30.2	(110)	
		26.3	(200)	
		5.1	alkyl halo	
	RT	57.7	(211)	Cub_bi_ (*Ia3d*)
		50.4	(220)	(a = 141 Å)
		37.7	(321)	
		35.2	(400)	
		33.3	Col_h_	
		31.5	(420)	
		30.2	(332)	
		27.7	(510)	
		5.0	alkyl halo	
**5**	RT	47.7	(100)	Col_h_ (a = 55.1 Å)
		27.1	(110)	
		5.0	alkyl halo	
**6**	140	52.8	(100)	Col_h_ (a = 61.0 Å)
		30.2	(110)	
		26.3	(200)	
		4.9	alkyl halo	

Note: alkyl halo determined from WAXS; all other peaks from MAXS.

## Data Availability

The data are contained within the article.

## Supplementary Materials

The following supporting information can be downloaded at: https://www.mdpi.com/article/10.3390/molecules29235633/s1. Figure S1: ^1^H NMR spectrum of compound **10**; Figure S2: ^13^C NMR spectrum of compound **10**; Figure S3: ^1^H-^1^H COSY NMR spectrum of **10**; Figure S4: ^1^H-^13^C HSQC NMR spectrum of **10**; Figure S5: ^1^H NMR spectrum of compound **3**; Figure S6: ^13^C NMR spectrum of compound **3**; Figure S7: ^1^H-^1^H COSY NMR spectrum of **3**; Figure S8: ^1^H-^13^C HSQC NMR spectrum of **3**; Figure S9: ^1^H NMR spectrum of compound **4**; Figure S10: ^13^C NMR spectrum of compound **4**; Figure S11: ^1^H-^1^H COSY NMR spectrum of **4**; Figure S12: ^1^H-^13^C HSQC NMR spectrum of **4**; Figure S13: ^1^H NMR spectrum of compound **15**; Figure S14: ^13^C NMR spectrum of compound **15**; Figure S15: ^1^H-^1^H COSY NMR spectrum of **15**; Figure S16: ^1^H-^13^C HSQC NMR spectrum of **15**; Figure S17: ^1^H NMR spectrum of compound **5**; Figure S18: ^13^C NMR spectrum of compound **5**; Figure S19: ^1^H-^1^H COSY NMR spectrum of **5**; Figure S20: ^1^H-^13^C HSQC NMR spectrum of **5**; Figure S21: ^1^H NMR spectrum of compound **6**; Figure S22: ^13^C NMR spectrum of compound **6**; Figure S23: ^1^H-^1^H COSY NMR spectrum of **6**; Figure S24: ^1^H-^13^C HSQC NMR spectrum of **6**; Figure S25: DSC thermogram of **3**; Figure S26: DSC thermogram of **4**; Figure S27: DSC thermogram of **5**; Figure S28: DSC thermogram of **6**; Figure S29: Cross-polarized micrographs of **3** during cooling cycle at 70.2 °C (a) and 35.7 °C (b), **4** during cooling cycle at 140.5 °C (c) and 48.3 °C (d), followed by standing at ambient temperature after several days (e), and reheating cycle to 67.1 °C (f), 150.0 °C (g), and. Finally, 160.3 °C (h), with five during cooling cycle at 35.2 °C (i), and six during cooling cycle at 143.0 °C (j) and 72.7 °C (k); Figure S30: XRD of **3**; Figure S31: XRD of **4**; Figure S32: XRD of **5**; Figure S33: XRD of **6**.
