# Supplementary material for: Synthesis and Mesomorphic Properties of Geometric and Conformation-Modulated Amphiphilic β-Cyclodextrin Liquid Crystals
Source: Molecules. 2024 Nov 28;29(23):5633. doi: 10.3390/molecules29235633 (PMC11643793; doi:10.3390/molecules29235633)
Supplement: Supplementary file 1 [file molecules-29-05633-s001.zip › molecules-3300144-supplementary.pdf]

Synthesis and Mesomorphic Properties of Geometric and Conformation-modulated Amphiphilic β-Cyclodextrin Liquid Crystals

Austin Che,[1] Homayoun Ghaseminezhad,[2] Carson O. Zellman,[2] Jessica Duong,[1] Vance E. Williams[2,*] and Chang-Chun Ling[1,*]

[1]Department of Chemistry, University of Calgary, Calgary, Alberta T2N 1N4, Canada

[2]Department of Chemistry, Simon Fraser University, Burnaby, British Columbia V5A 1S6, Canada

Corresponding author: Tel.: +1-403-220-2768; fax: +1-403-289-9488;
e-mail: ccling@ucalgary.ca

Contents

NMR Spectra 4

NMR Spectra of compound **10** 4

Figure S1. ^{1}H NMR Spectrum of compound **10**. 4

Figure S2. ^{13}C NMR Spectrum of compound **10** 5

Figure S3. ^{1}H-^{1}H COSY NMR Spectrum of **10** 6

Figure S4. ^{1}H-^{13}C HSQC NMR Spectrum of **10** 7

NMR Spectra of compound **3** 8

Figure S5. ^{1}H NMR Spectrum of compound **3**. 8

Figure S6. ^{13}C NMR Spectrum of compound **3** 9

Figure S7. ^{1}H-^{1}H COSY NMR Spectrum of **3** 10

Figure S8. ^{1}H-^{13}C HSQC NMR Spectrum of **3** 11

NMR Spectra of compound **4** 12

Figure S9. ^{1}H NMR Spectrum of compound **4**. 12

Figure S10. ^{13}C NMR Spectrum of compound **4** 13

Figure S11. ^{1}H-^{1}H COSY NMR Spectrum of **4** 14

Figure S12. ^{1}H-^{13}C HSQC NMR Spectrum of **4** 15

NMR Spectra of compound **15** 16

Figure S13. ^{1}H NMR Spectrum of compound **15**. 16

Figure S14. ^{13}C NMR Spectrum of compound **15** 17

Figure S15. ^{1}H-^{1}H COSY NMR Spectrum of **15** 18

Figure S16. ^{1}H-^{13}C HSQC NMR Spectrum of **15** 19

NMR Spectra of compound **5** 20

Figure S17. ^{1}H NMR Spectrum of compound **5**. 20

Figure S18. ^{13}C NMR Spectrum of compound **5** 21

Figure S19. ^{1}H-^{1}H COSY NMR Spectrum of **5** 22

Figure S20. ^{1}H-^{13}C HSQC NMR Spectrum of **5** 23

NMR Spectra of compound **6** 24

Figure S21. ^{1}H NMR Spectrum of compound **6**. 24

Figure S22. ^{13}C NMR Spectrum of compound **6** 25

Figure S23. ^{1}H-^{1}H COSY NMR Spectrum of **6** 26

Figure S24. ^{1}H-^{13}C HSQC NMR Spectrum of **6** 27

Differential Scanning Calorimetry (DSC) 28

Figure S25. DSC Thermogram of **3** 28

Figure S26. DSC Thermogram of **4** 29

Figure S27. DSC Thermogram of **5** 30

Figure S28. DSC Thermogram of **6** 31

Figure S29. Cross-polarized micrographs of **3** during cooling cycle at 70.2 °C (**a**) and 35.7 °C (**b**), **4** during cooling cycle at 140.5 °C (**c**) and 48.3 °C (**d**), then standing at ambient temperature after several days (**e**), and reheating cycle to 67.1 °C (**f**), 150.0 °C (**g**) and finally at 160.3 °C (**h**), **5** during cooling cycle at 35.2 °C (**i**), **6** during cooling cycle at 143.0 °C (**j**) and 72.7 °C (**k**). 32

X-Ray Diffraction (XRD) 33

Figure S30. XRD of **3** 33

Figure S31. XRD of **4** 34

Figure S32. XRD of **5** 35

Figure S33. XRD of **6** 36

NMR Spectra

NMR Spectra of compound **10**

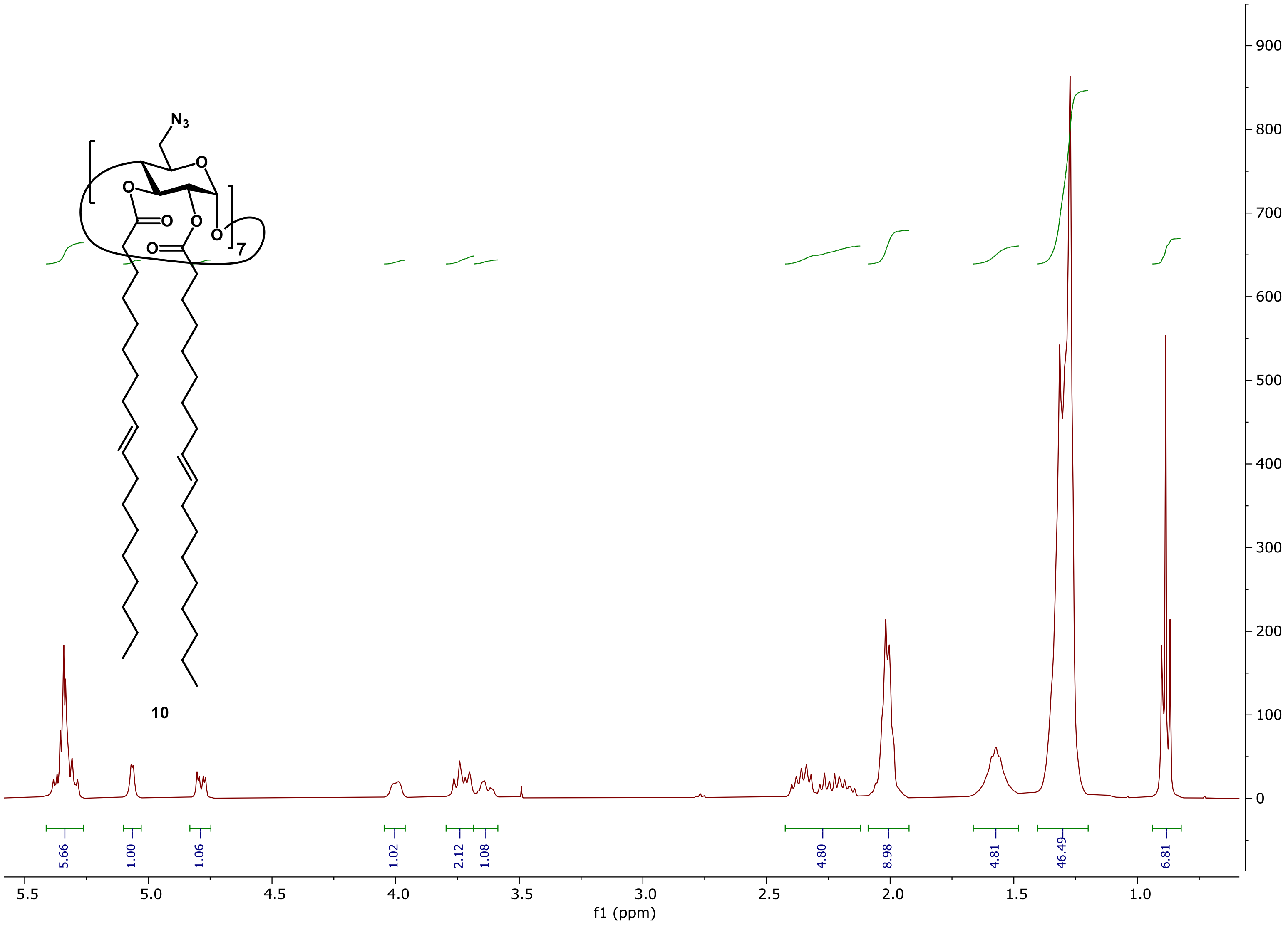

Figure S1. ^{1}H NMR Spectrum of compound **10**

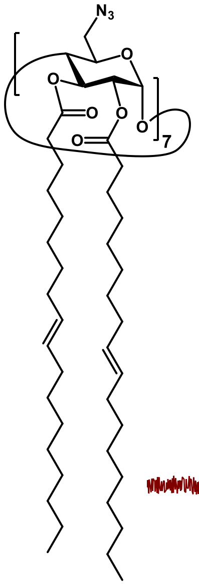

10

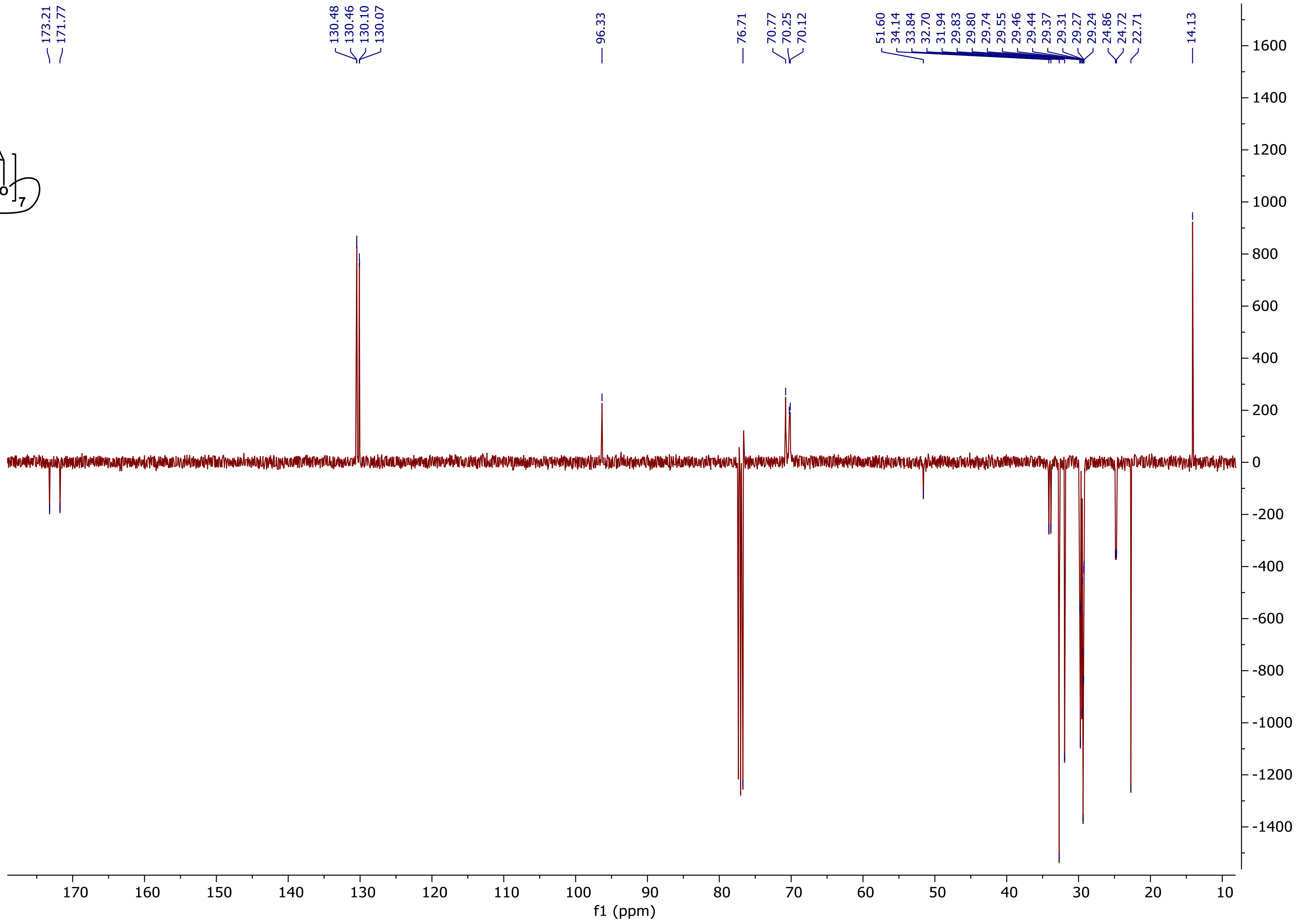

Figure S2. ^{13}C NMR Spectrum of compound **10**

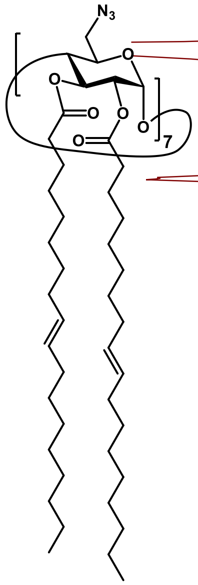

10

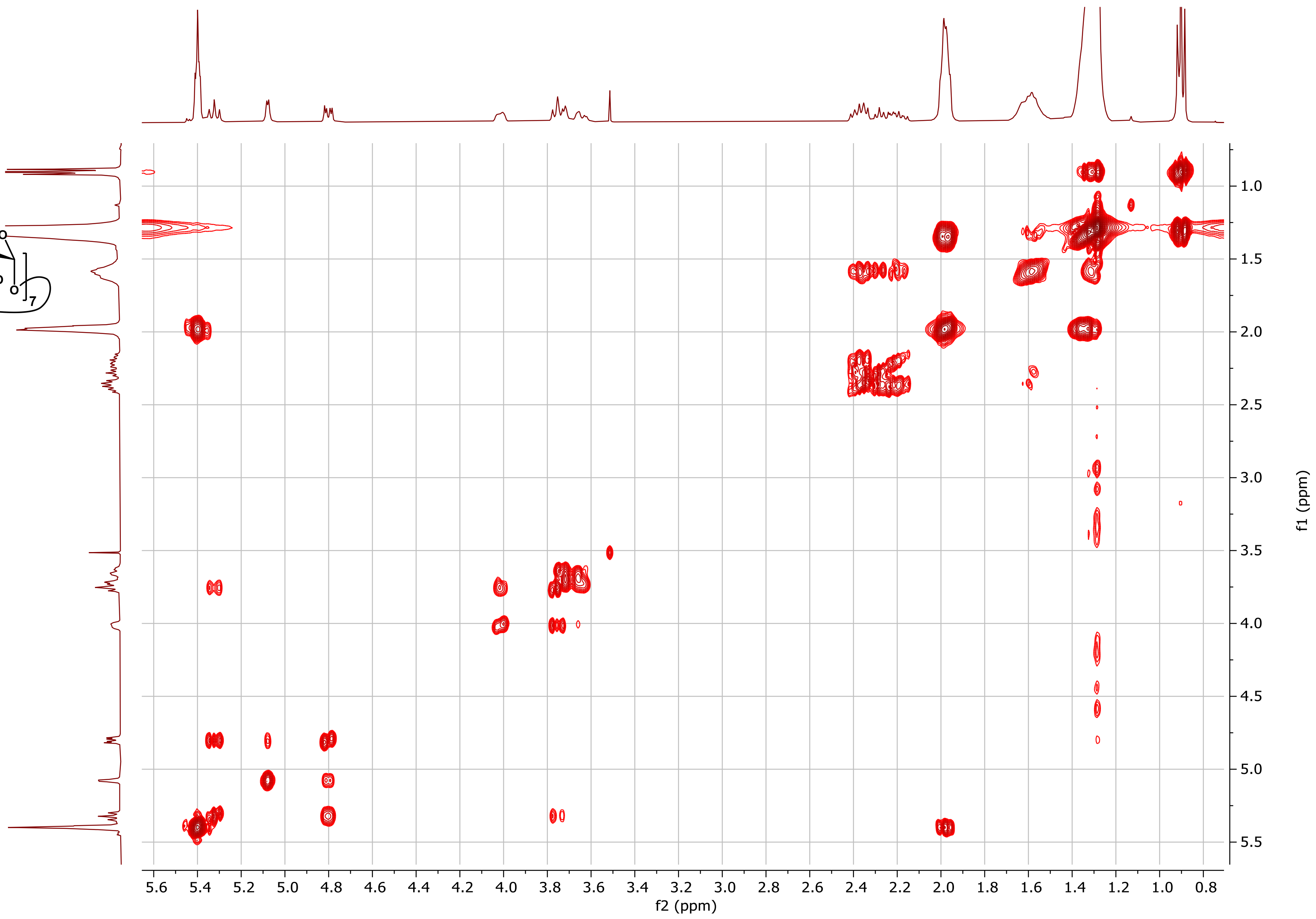

Figure S3. ^{1}H-^{1}H COSY NMR Spectrum of **10**

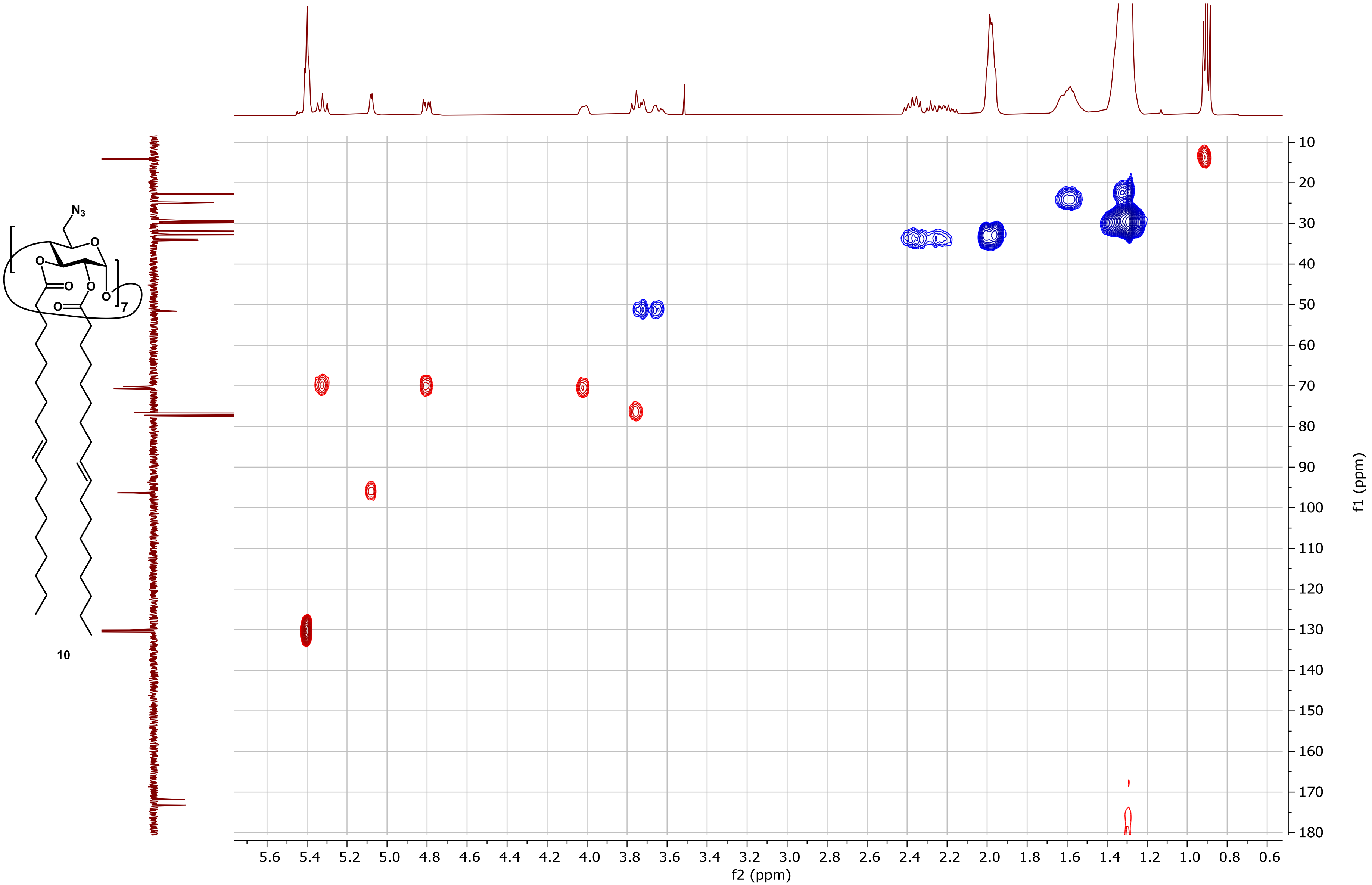

Figure S4. $^{1}H-^{13}C$ HSQC NMR Spectrum of **10**

NMR Spectra of compound **3**

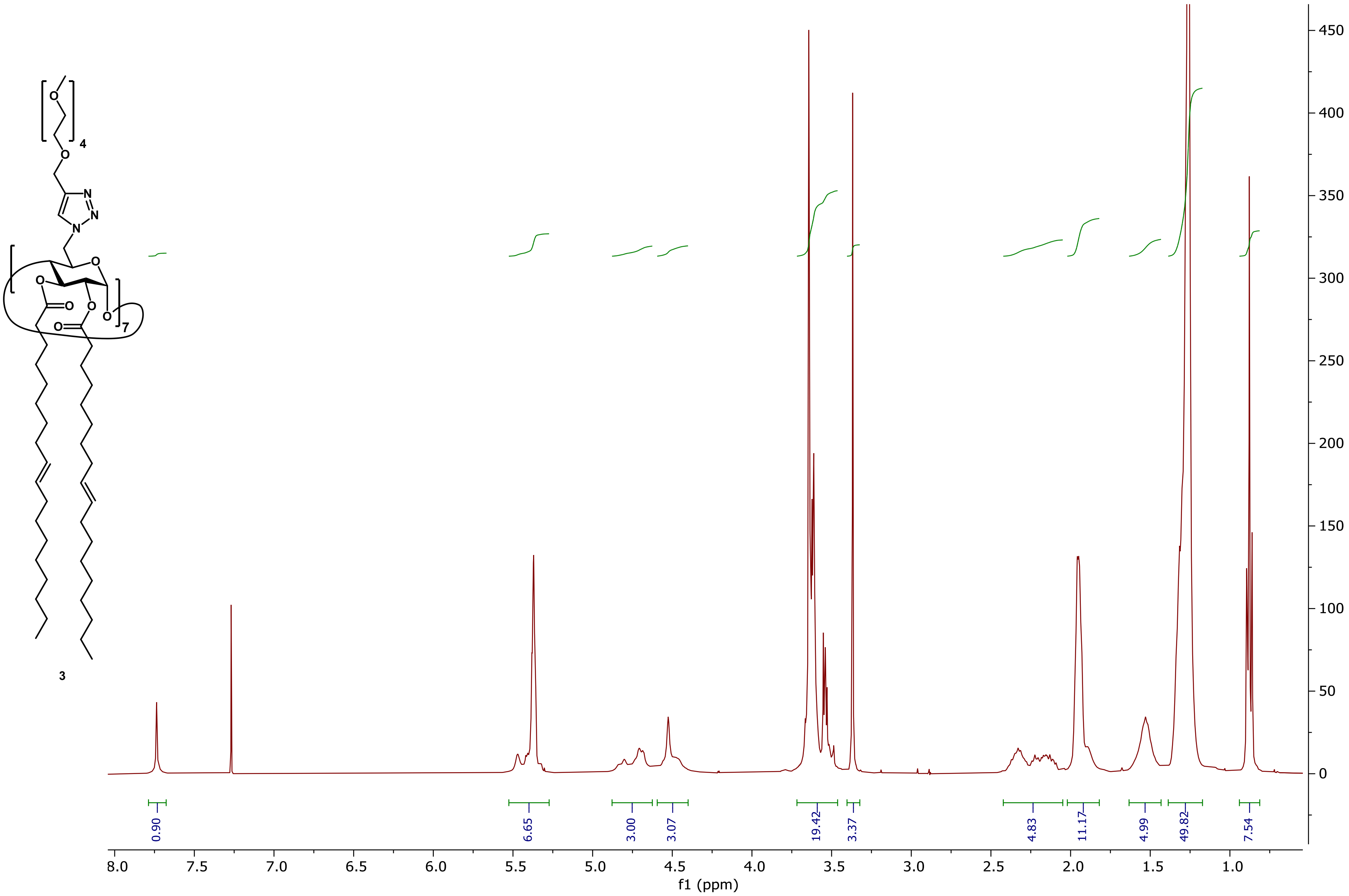

Figure S5. ^{1}H NMR Spectrum of compound **3**

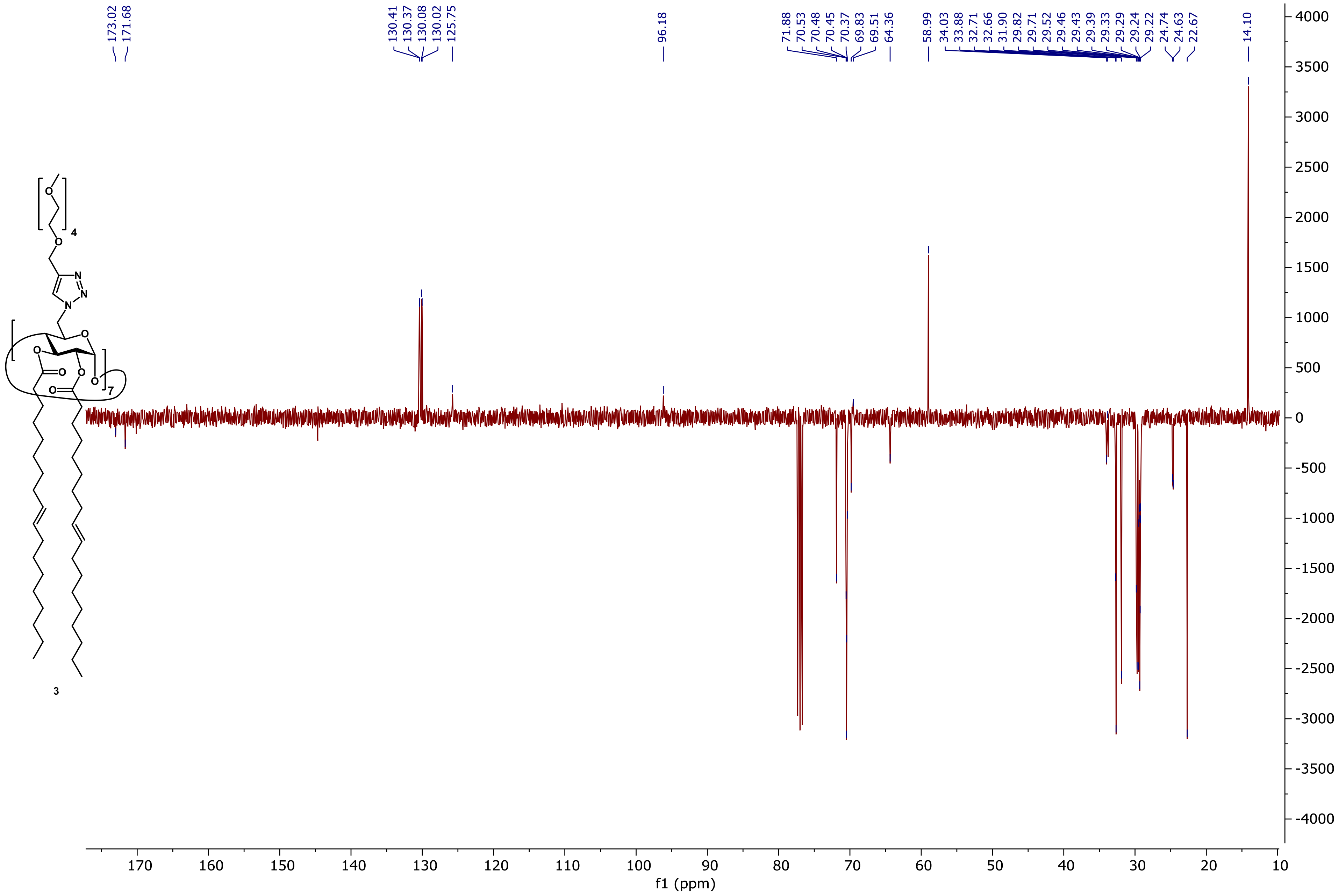

Figure S6. ^{13}C NMR Spectrum of compound **3**

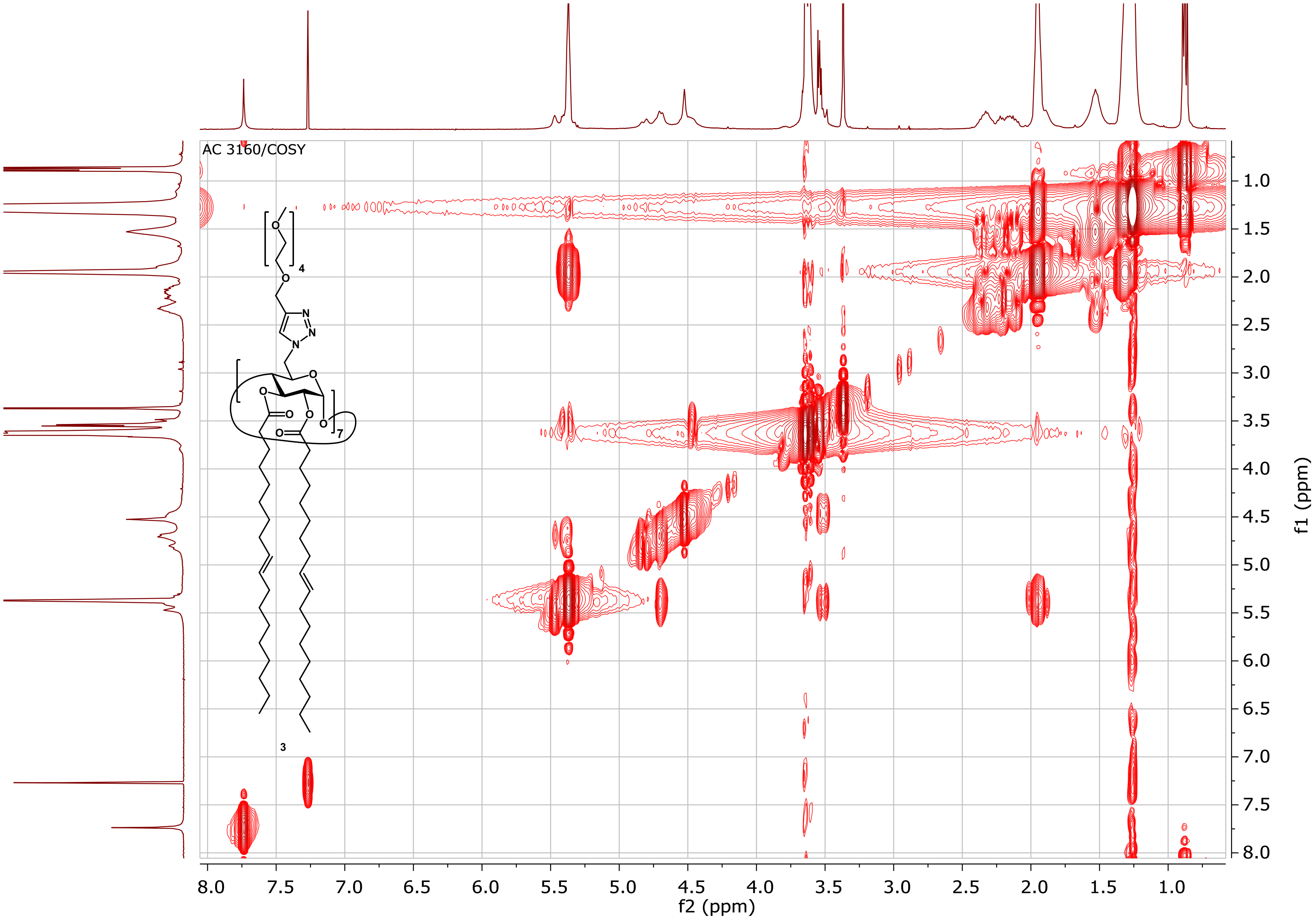

Figure S7. ^{1}H-^{1}H COSY NMR Spectrum of **3**

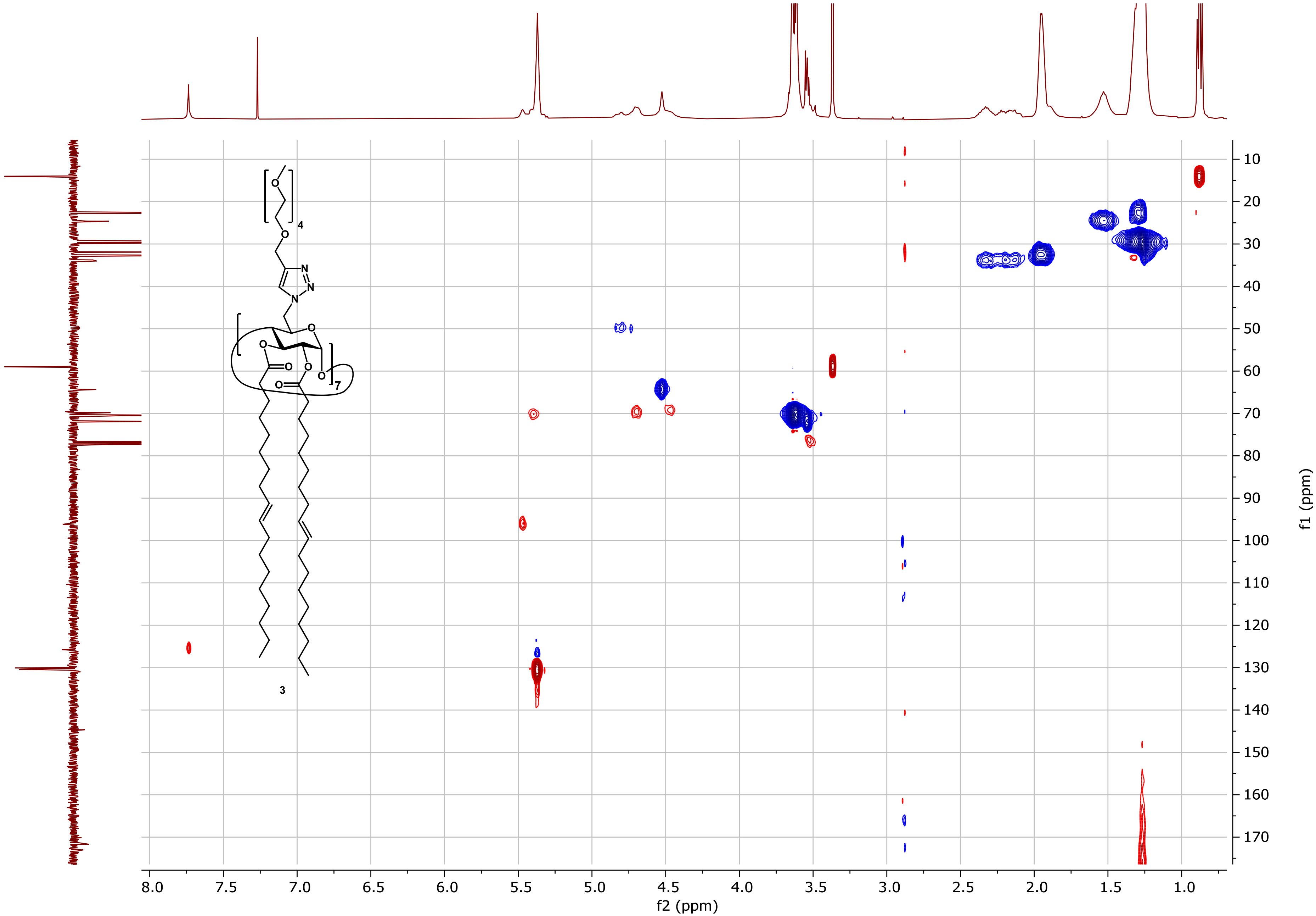

Figure S8. ^{1}H-^{13}C HSQC NMR Spectrum of **3**

NMR Spectra of compound **4**

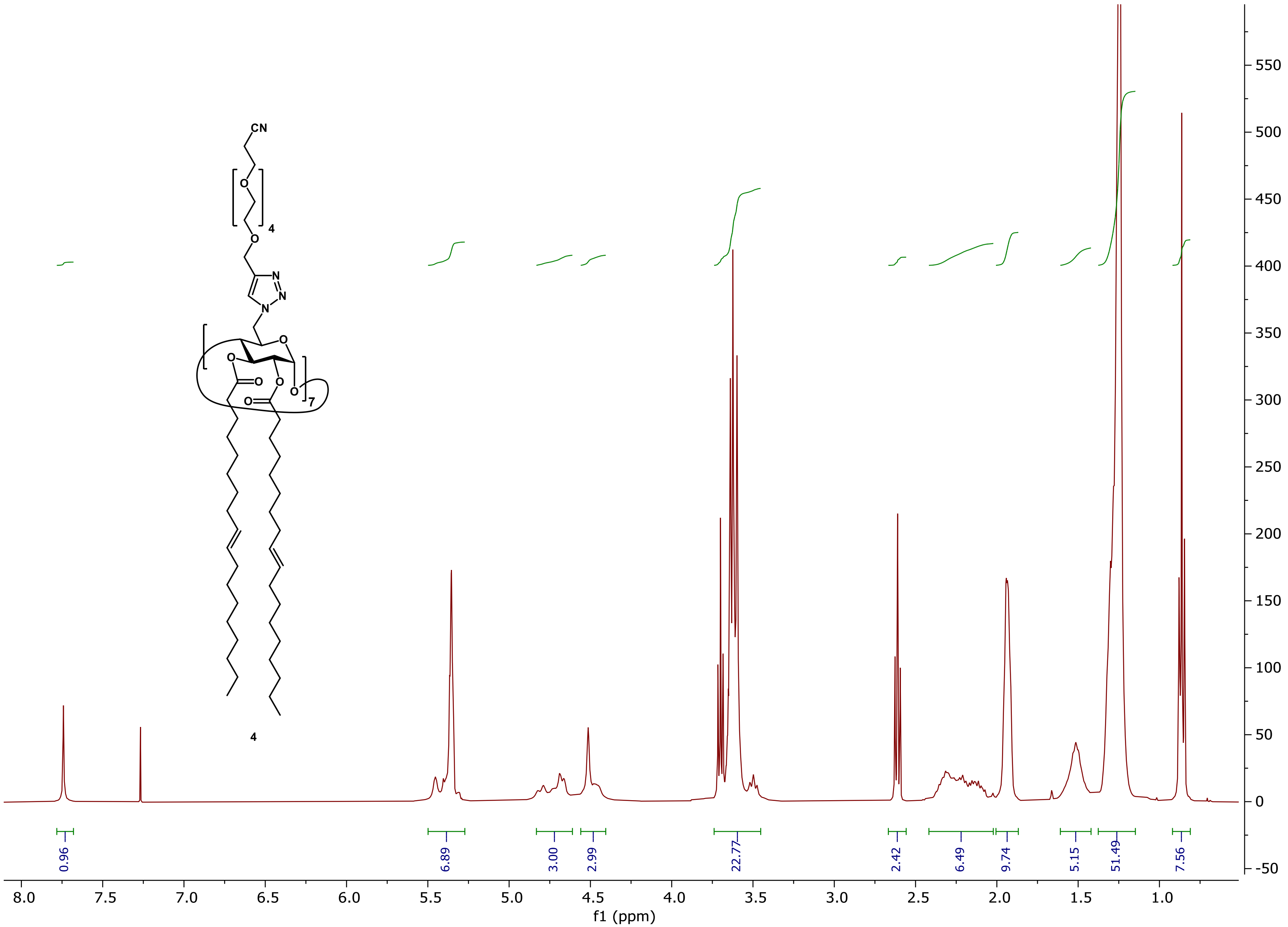

Figure S9. ^{1}H NMR Spectrum of compound **4**

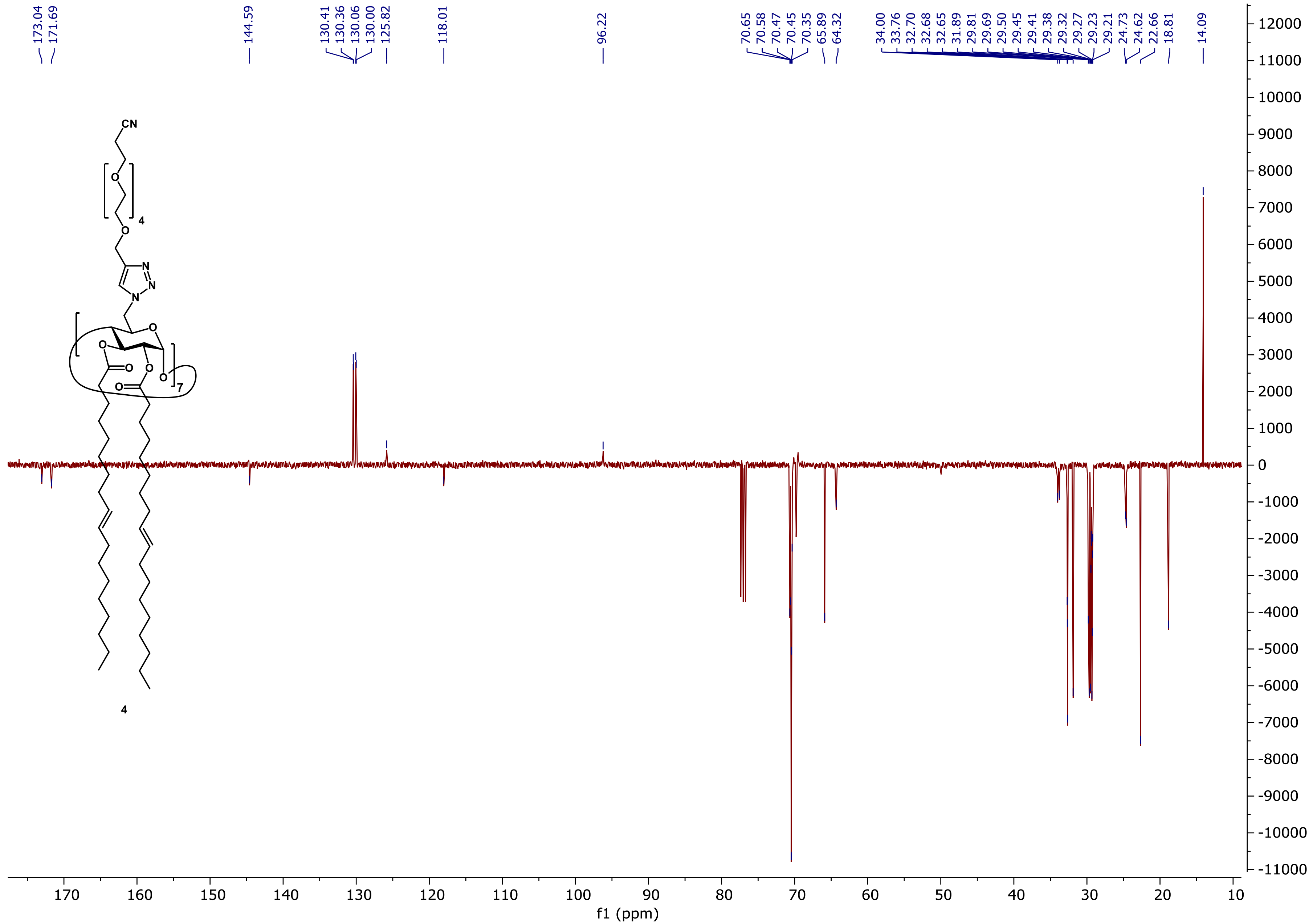

Figure S10. ^{13}C NMR Spectrum of compound **4**

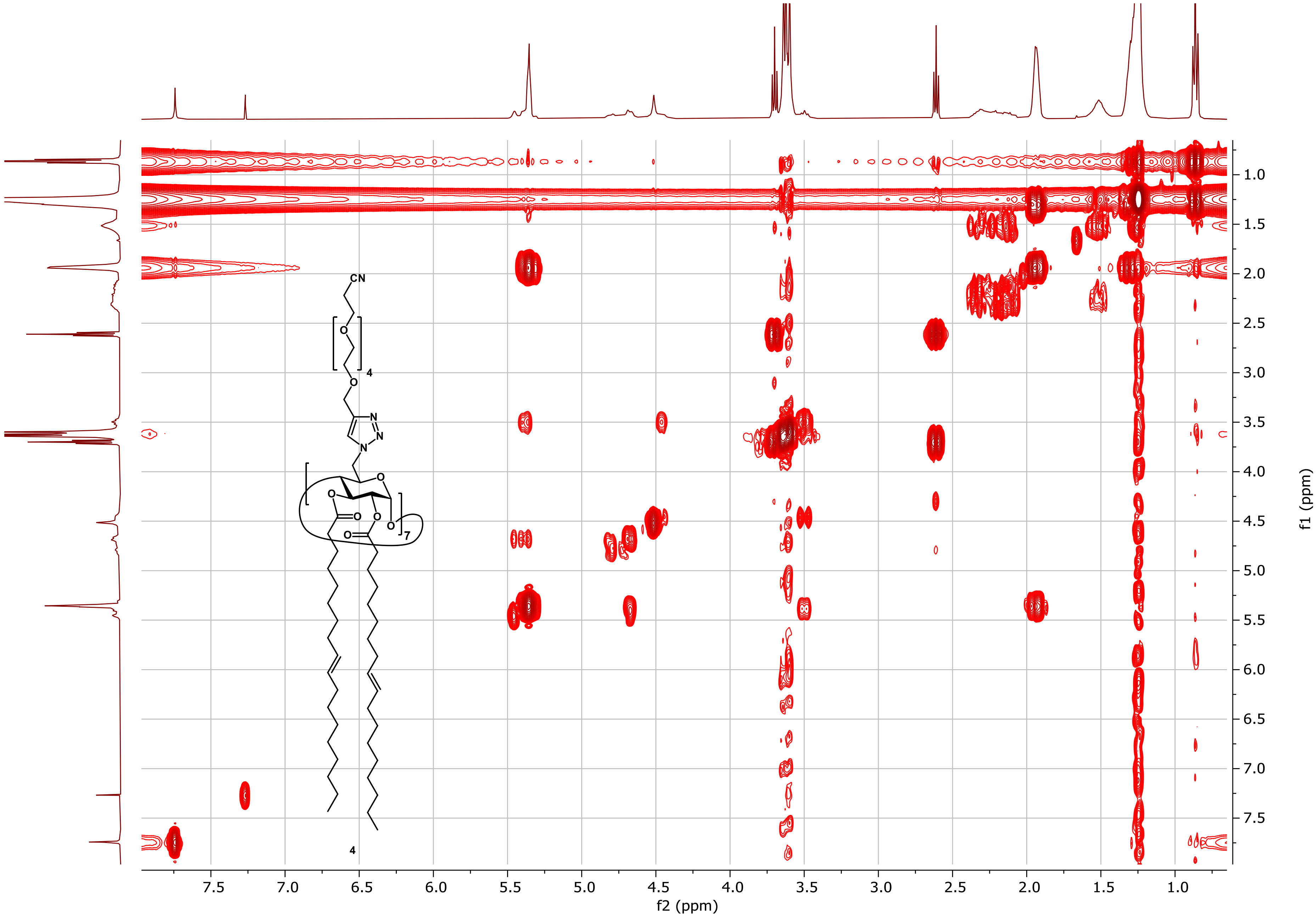

Figure S11. ^{1}H-^{1}H COSY NMR Spectrum of **4**

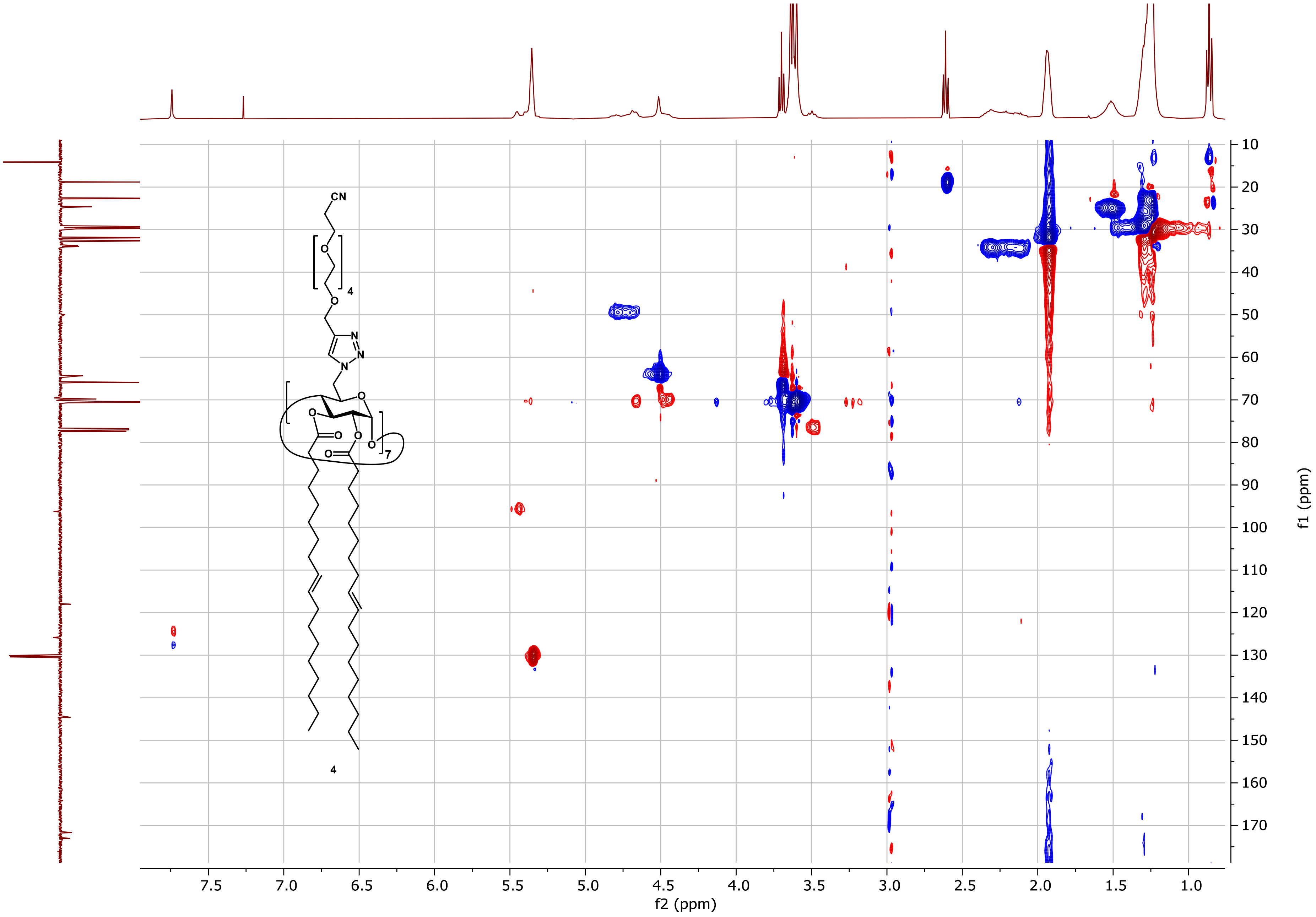

Figure S12. ^{1}H-^{13}C HSQC NMR Spectrum of **4**

NMR Spectra of compound **15**

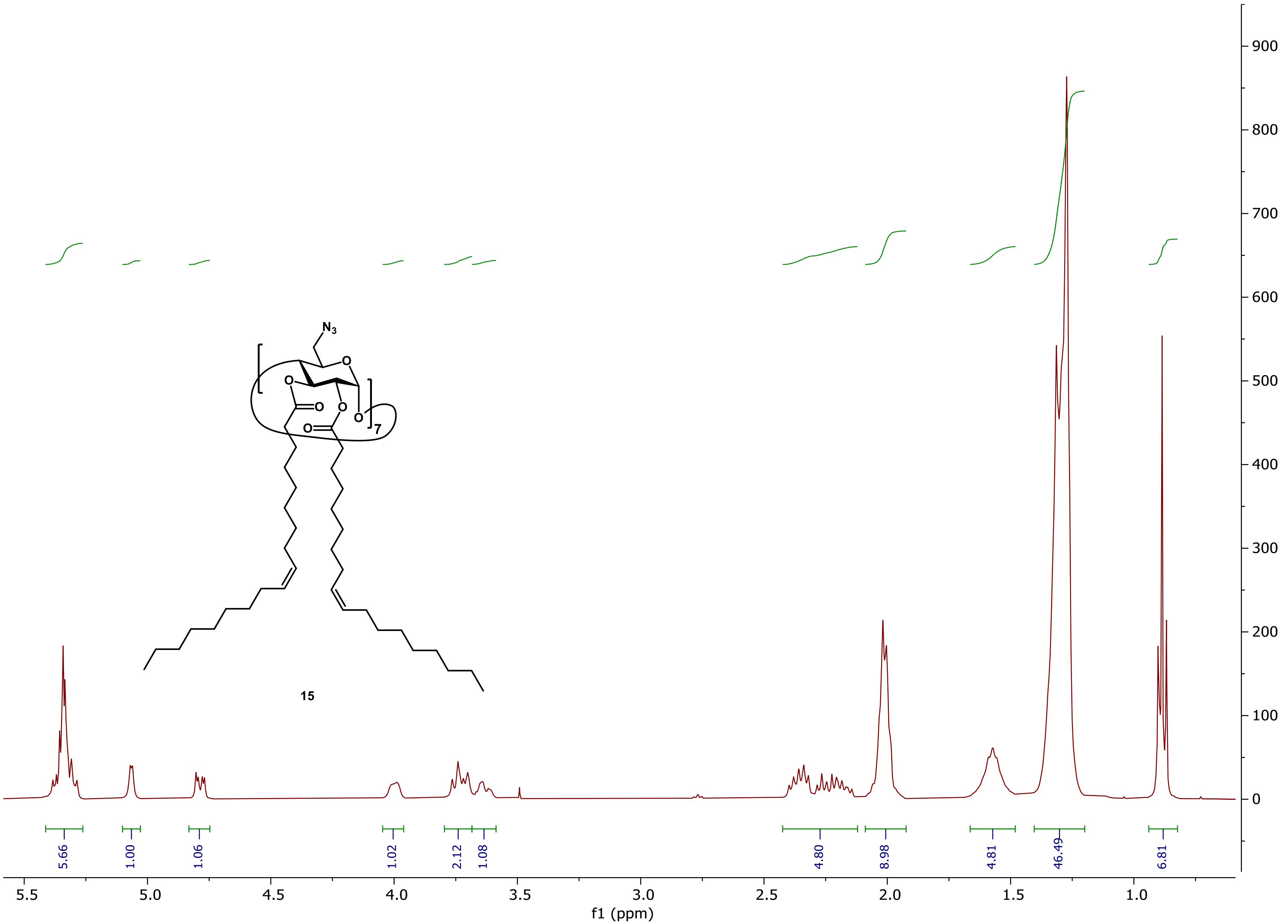

Figure S13. ^{1}H NMR Spectrum of compound **15**

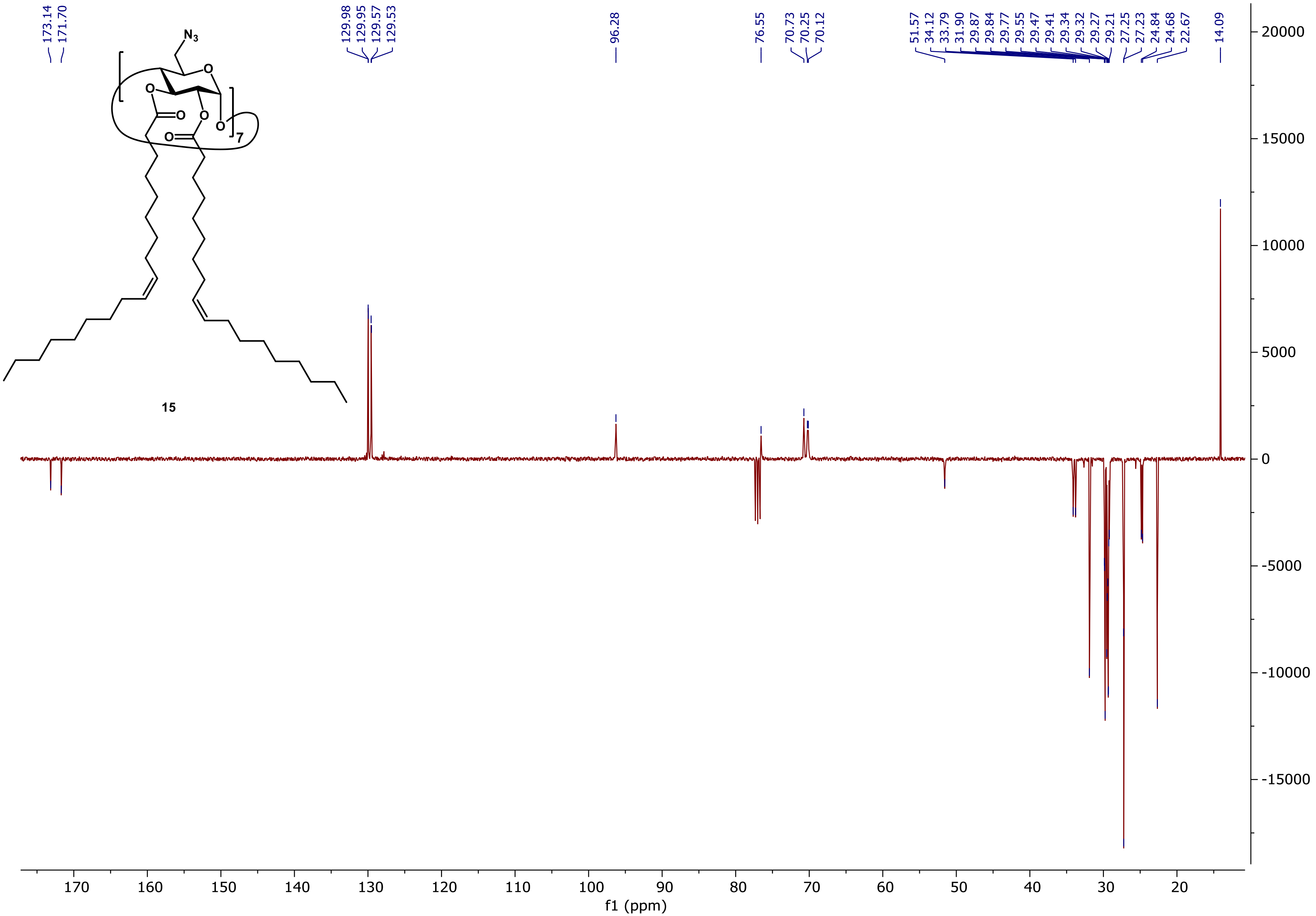

Figure S14. ^{13}C NMR Spectrum of compound **15**

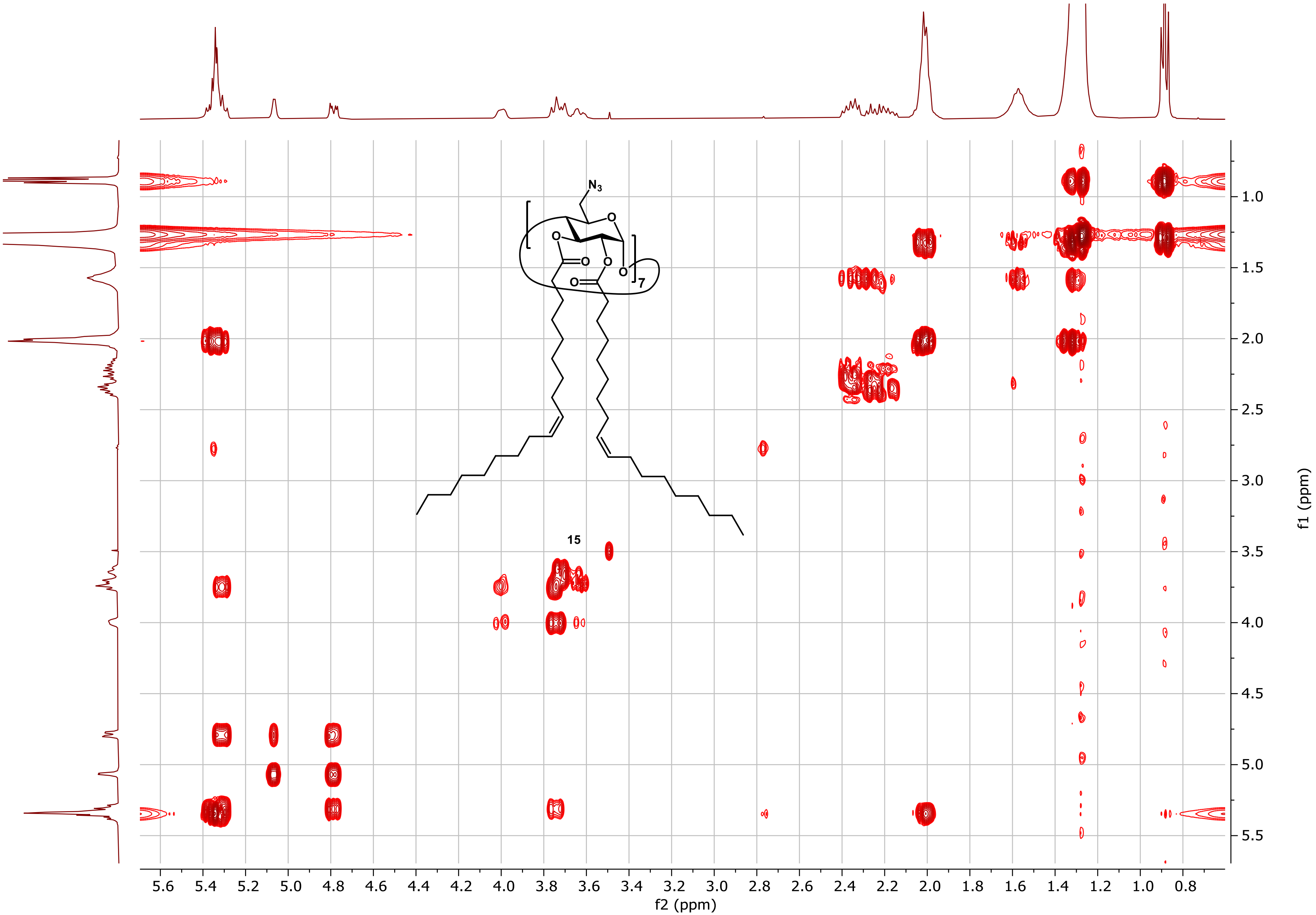

Figure S15. ^{1}H-^{1}H COSY NMR Spectrum of **15**

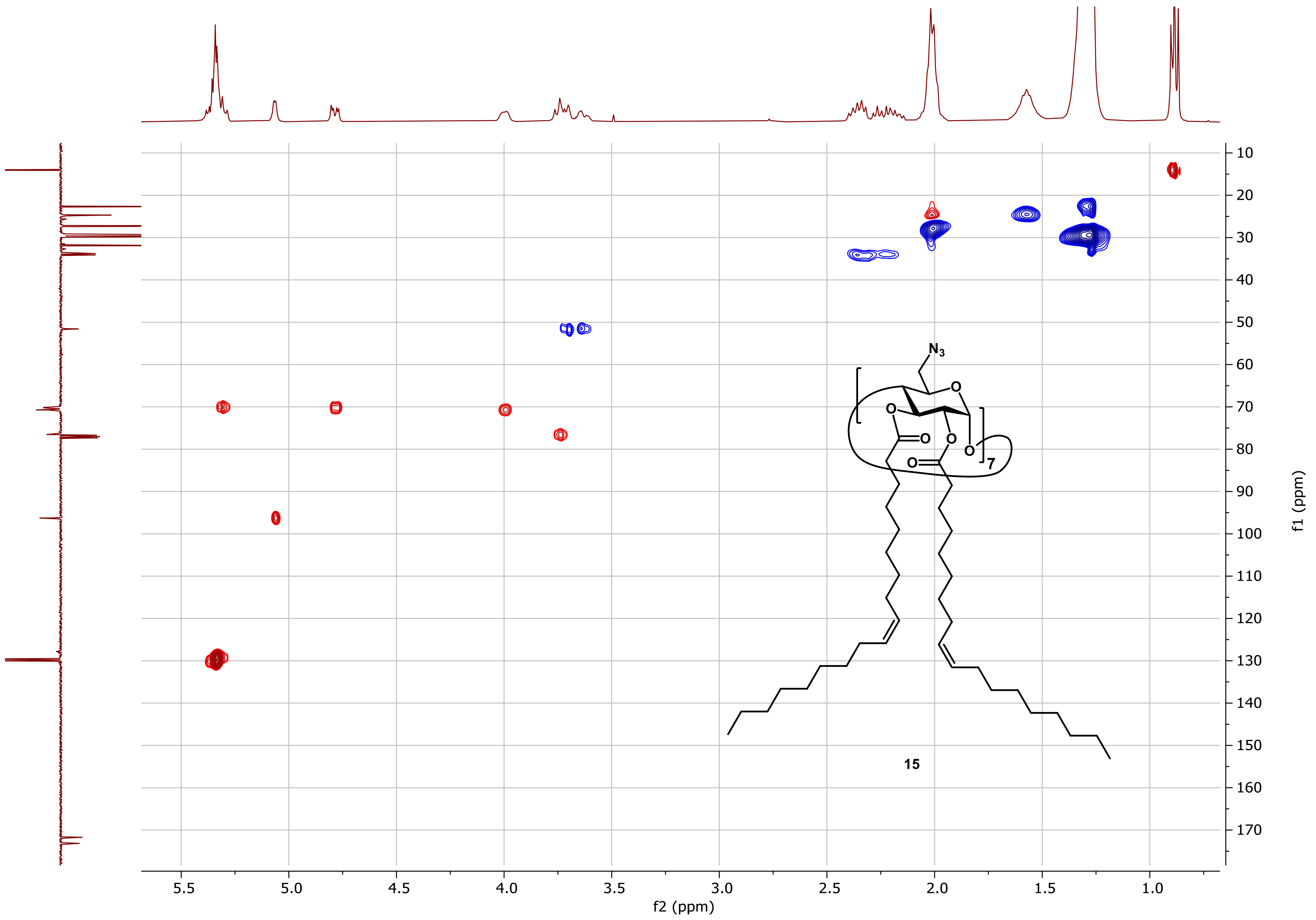

Figure S16. ^{1}H-^{13}C HSQC NMR Spectrum of **15**

NMR Spectra of compound **5**

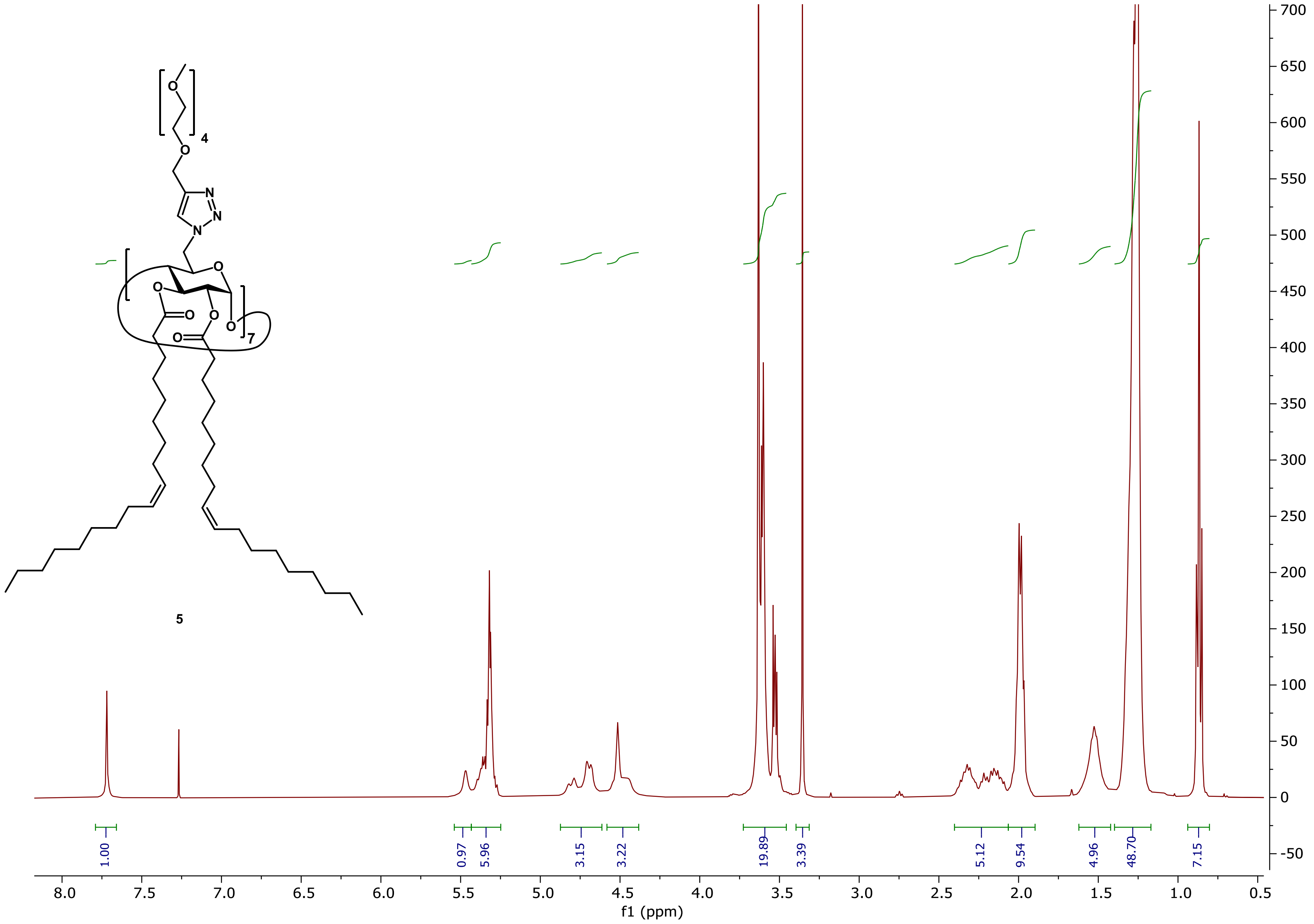

Figure S17. ^{1}H NMR Spectrum of compound **5**

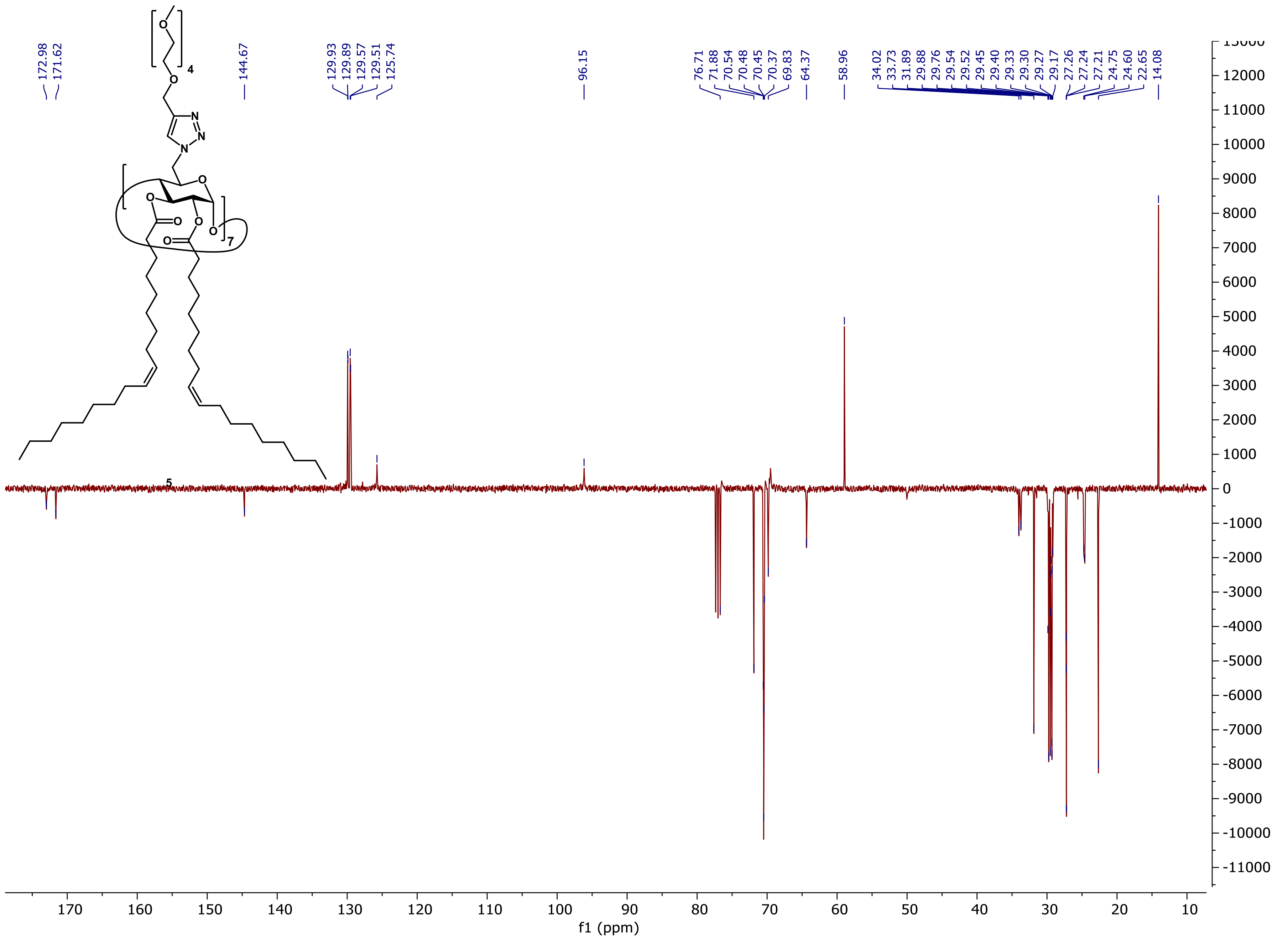

Figure S18. ^{13}C NMR Spectrum of compound **5**

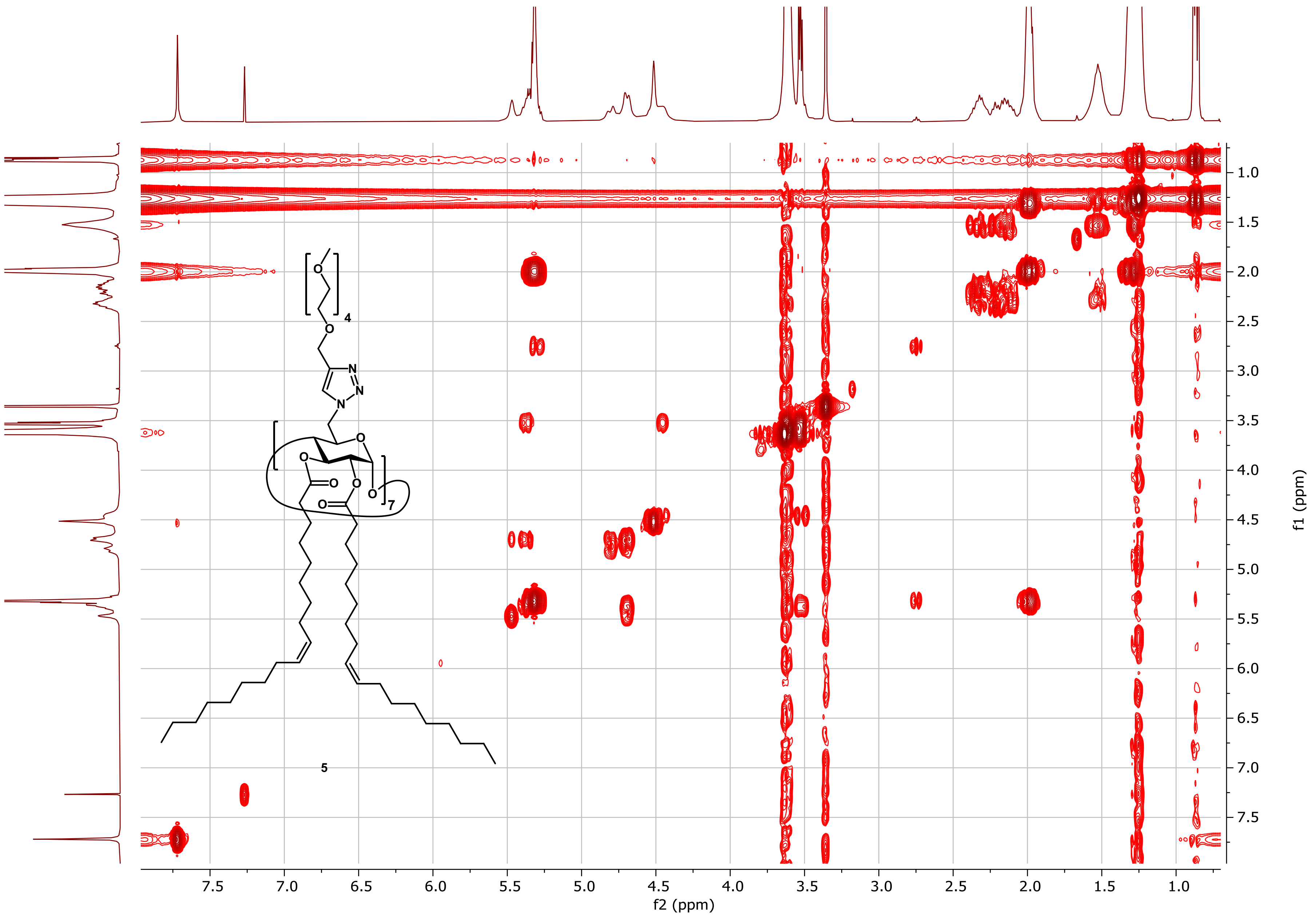

Figure S19. ^{1}H-^{1}H COSY NMR Spectrum of **5**

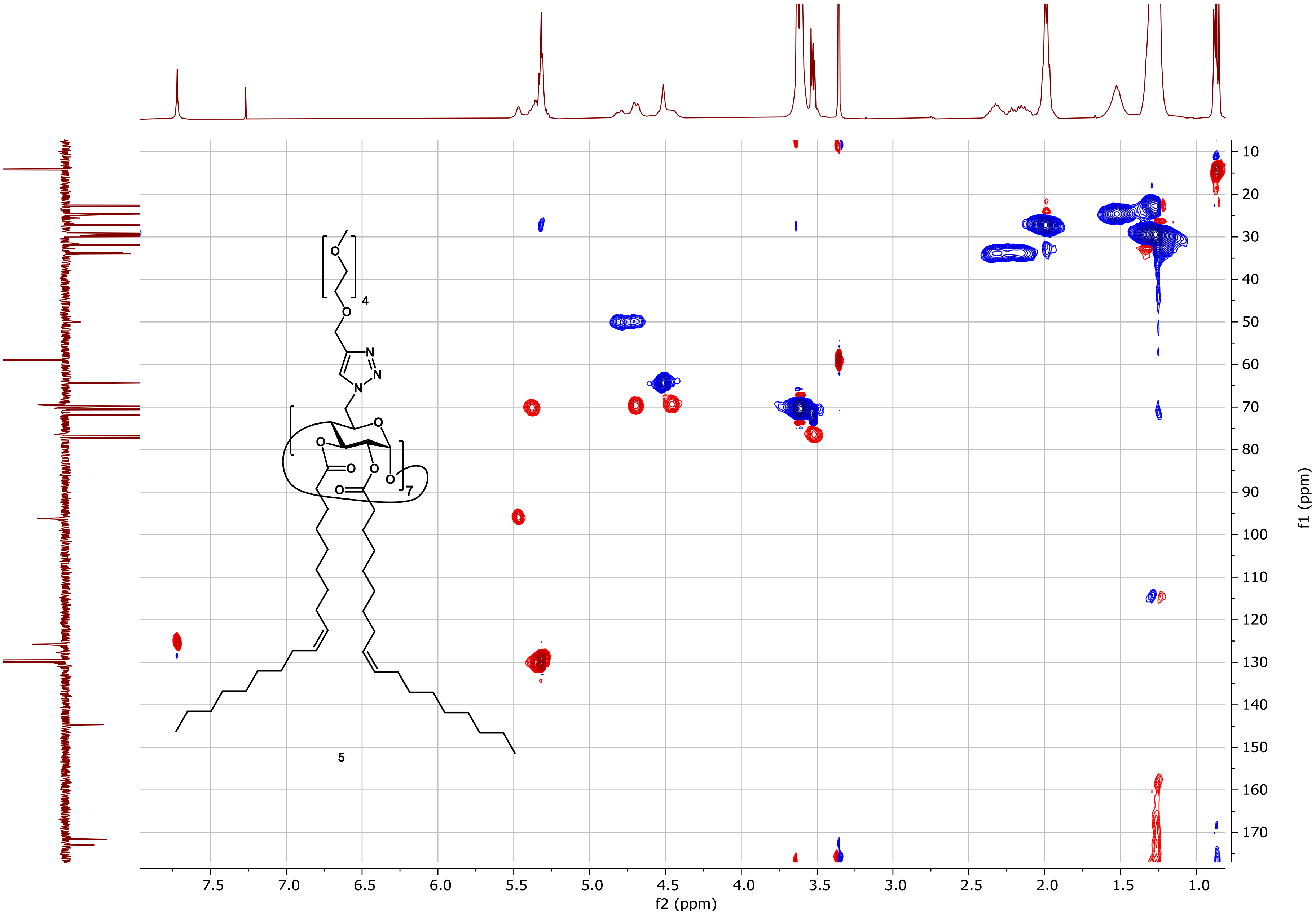

Figure S20. ^{1}H-^{13}C HSQC NMR Spectrum of **5**

NMR Spectra of compound **6**

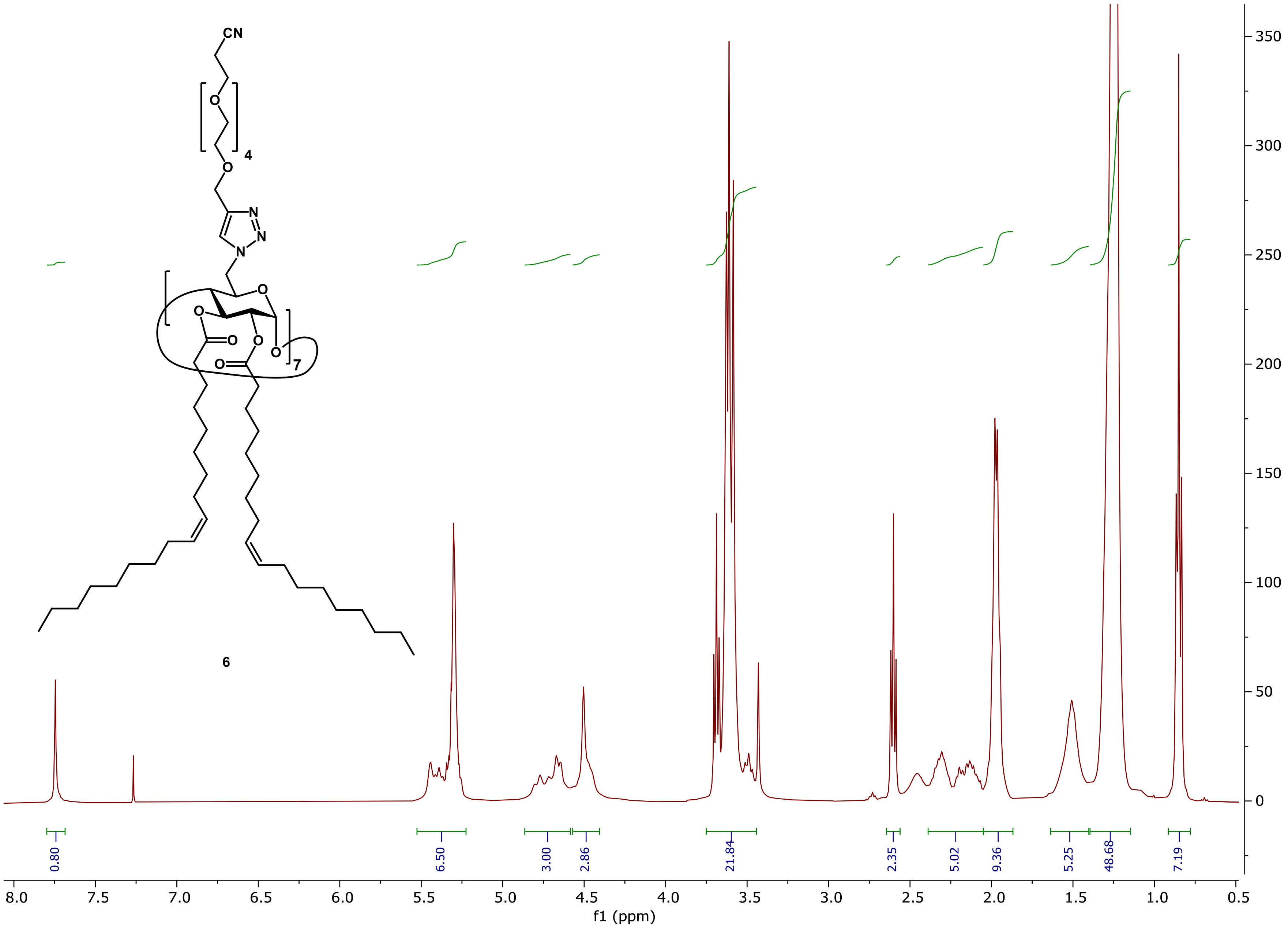

Figure S21. ^{1}H NMR Spectrum of compound **6**

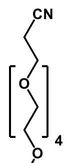

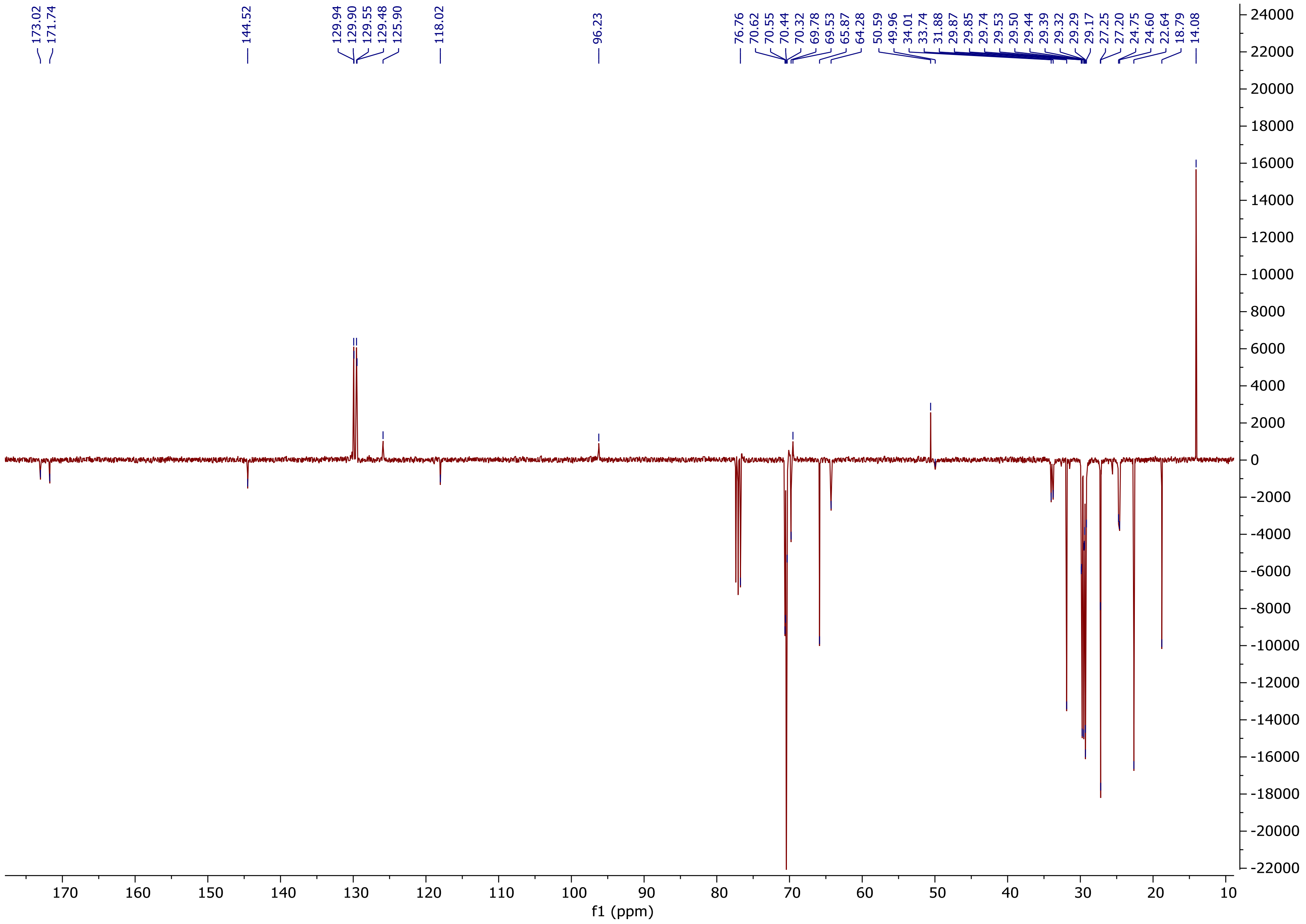

Figure S22. ^{13}C NMR Spectrum of compound **6**

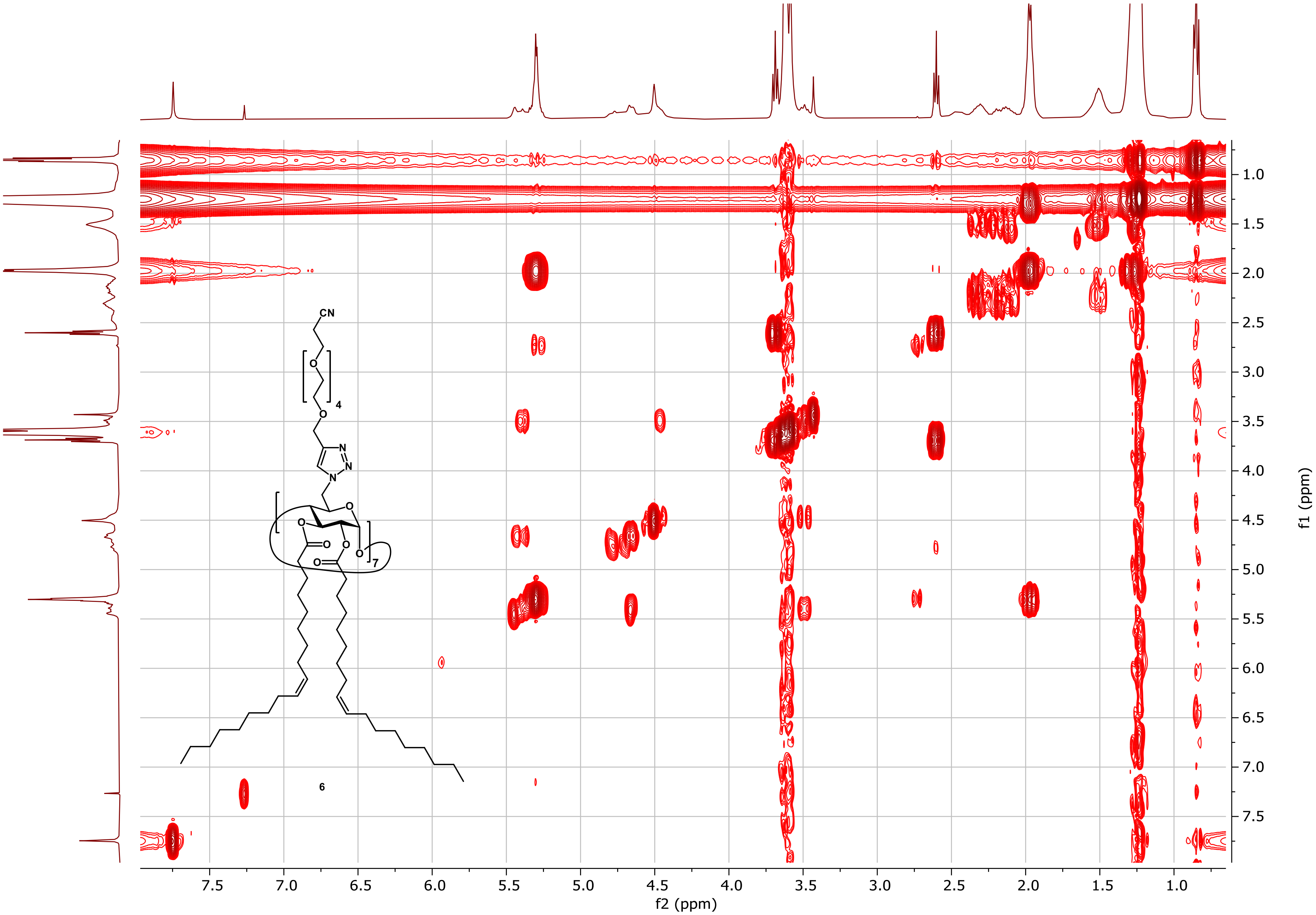

Figure S23. ¹H-¹H COSY NMR Spectrum of **6**

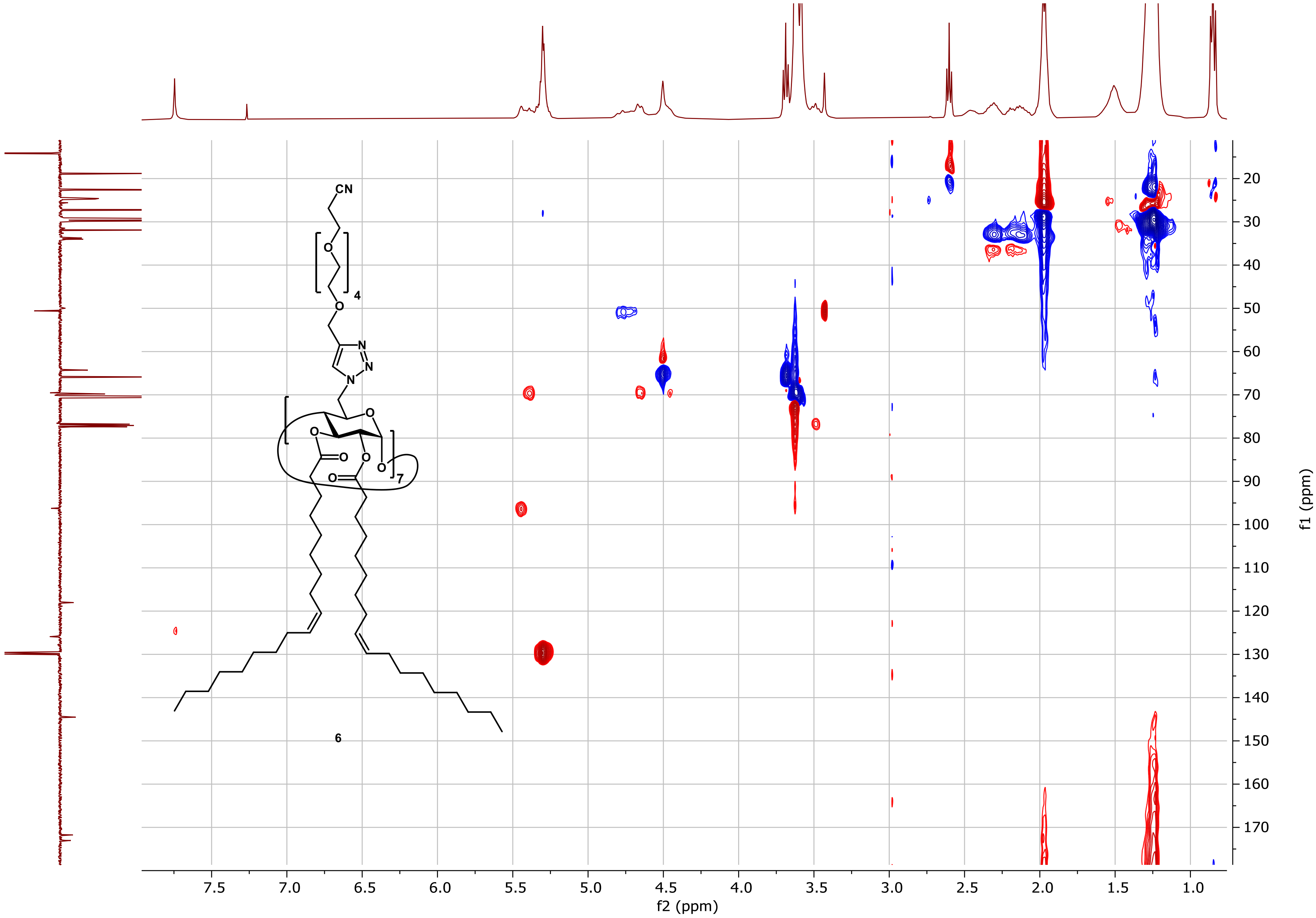

Figure S24. ^{1}H-^{13}C HSQC NMR Spectrum of **6**

Differential Scanning Calorimetry (DSC)

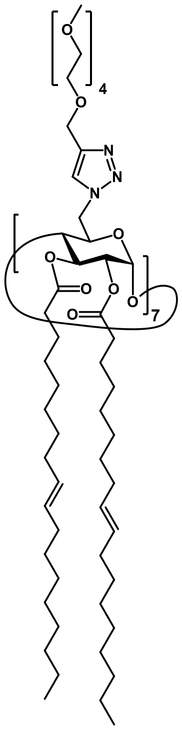

3

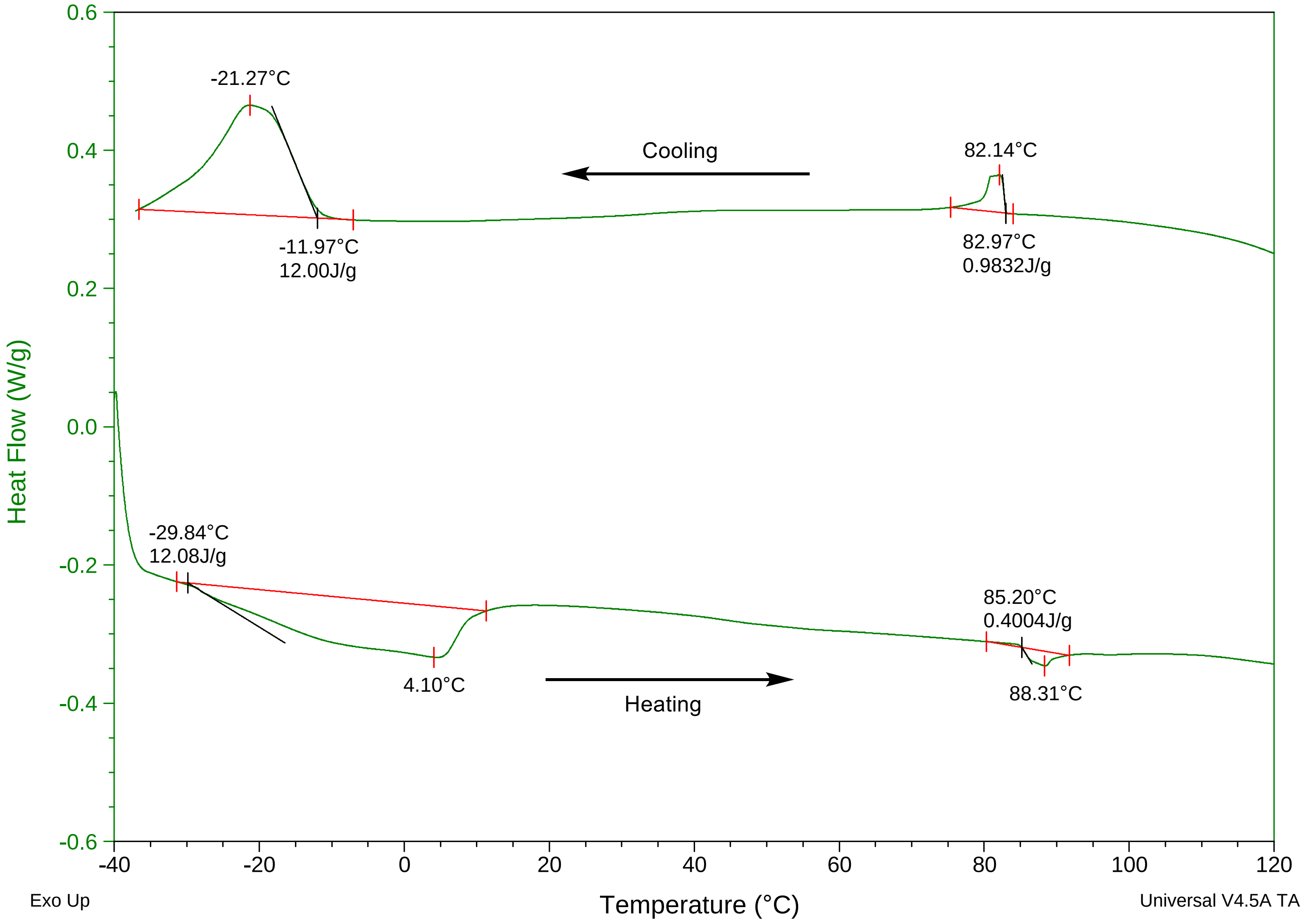

Figure S25. DSC Thermogram of **3**

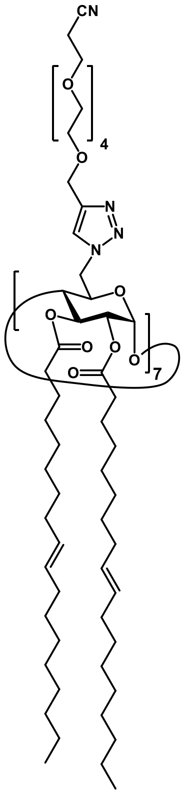

4

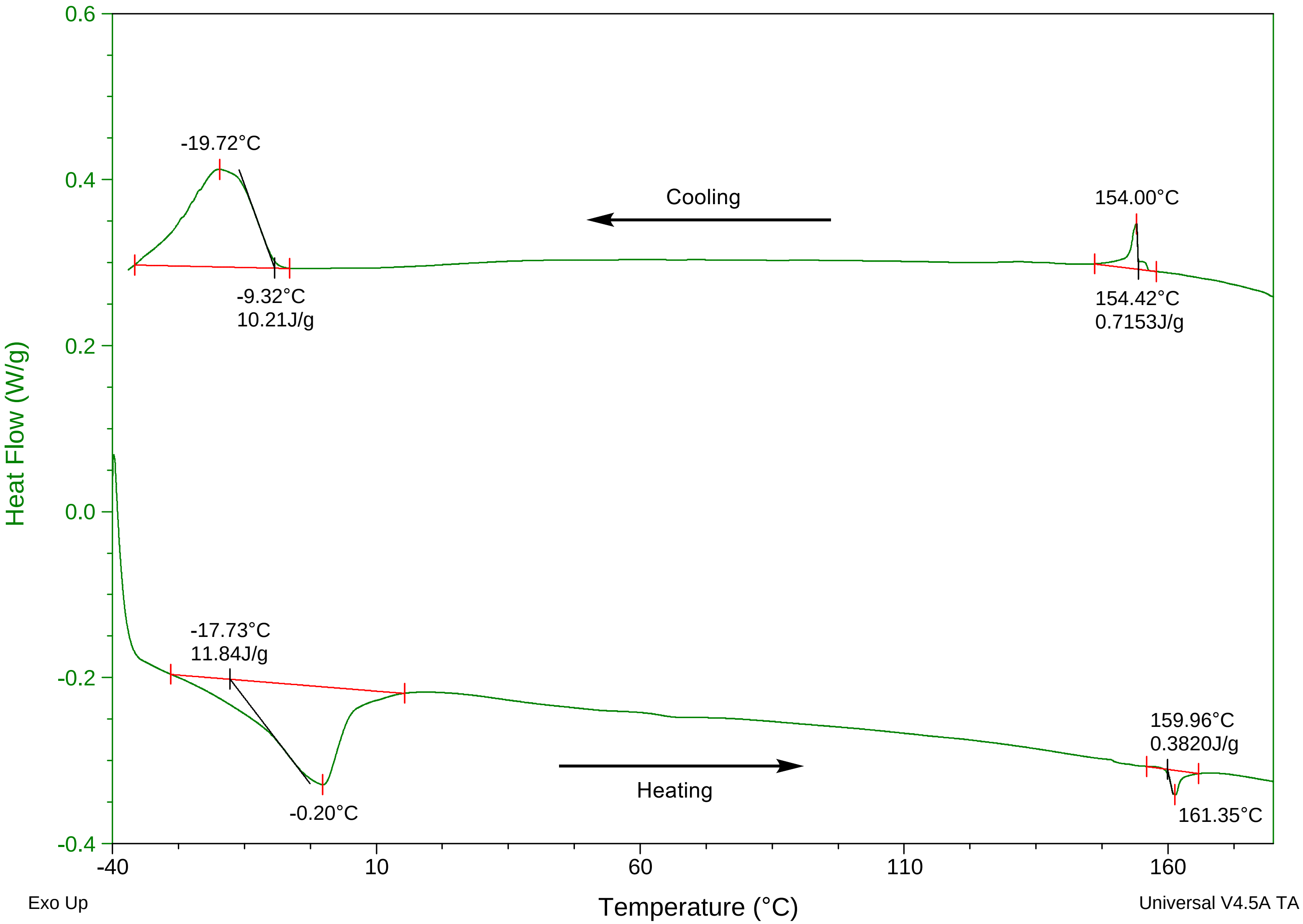

Figure S26. DSC Thermogram of **4**

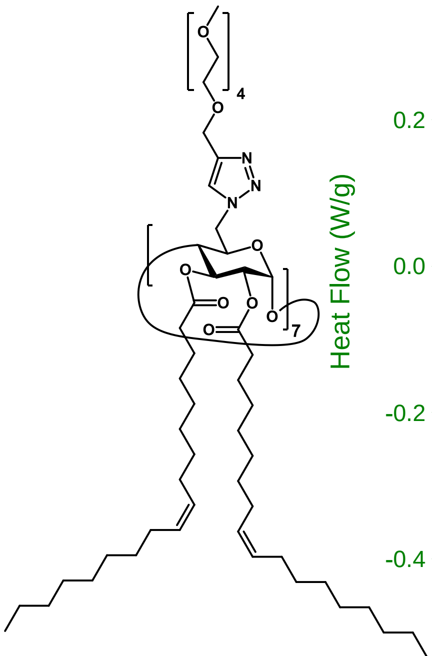

5

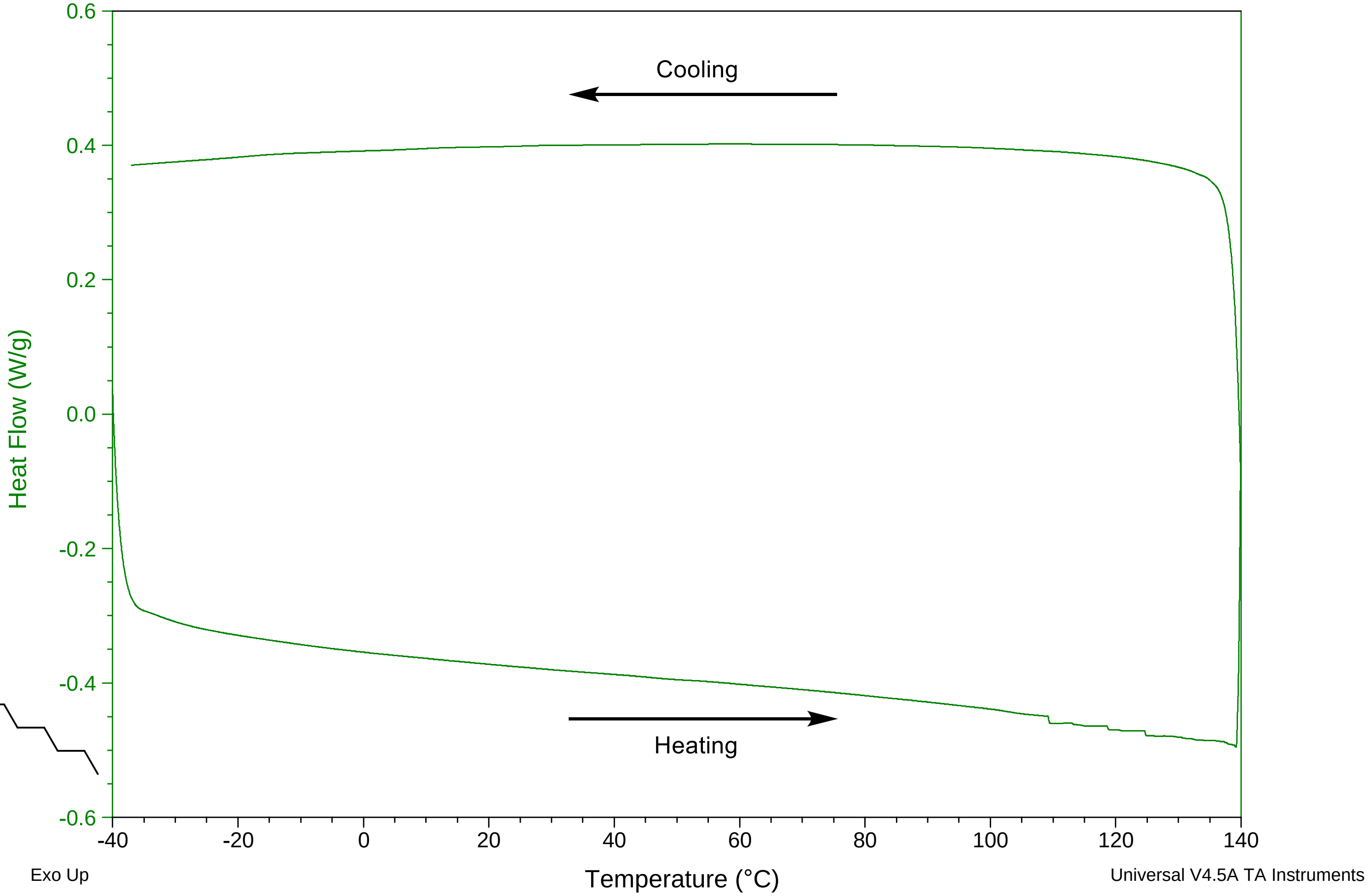

Figure S27. DSC Thermogram of **5**

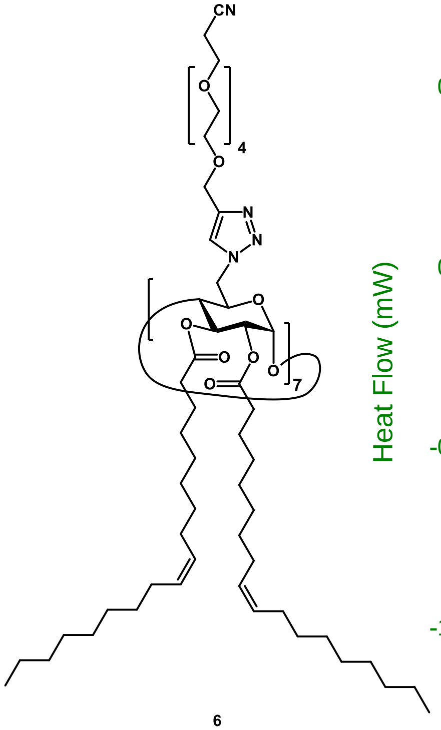

6

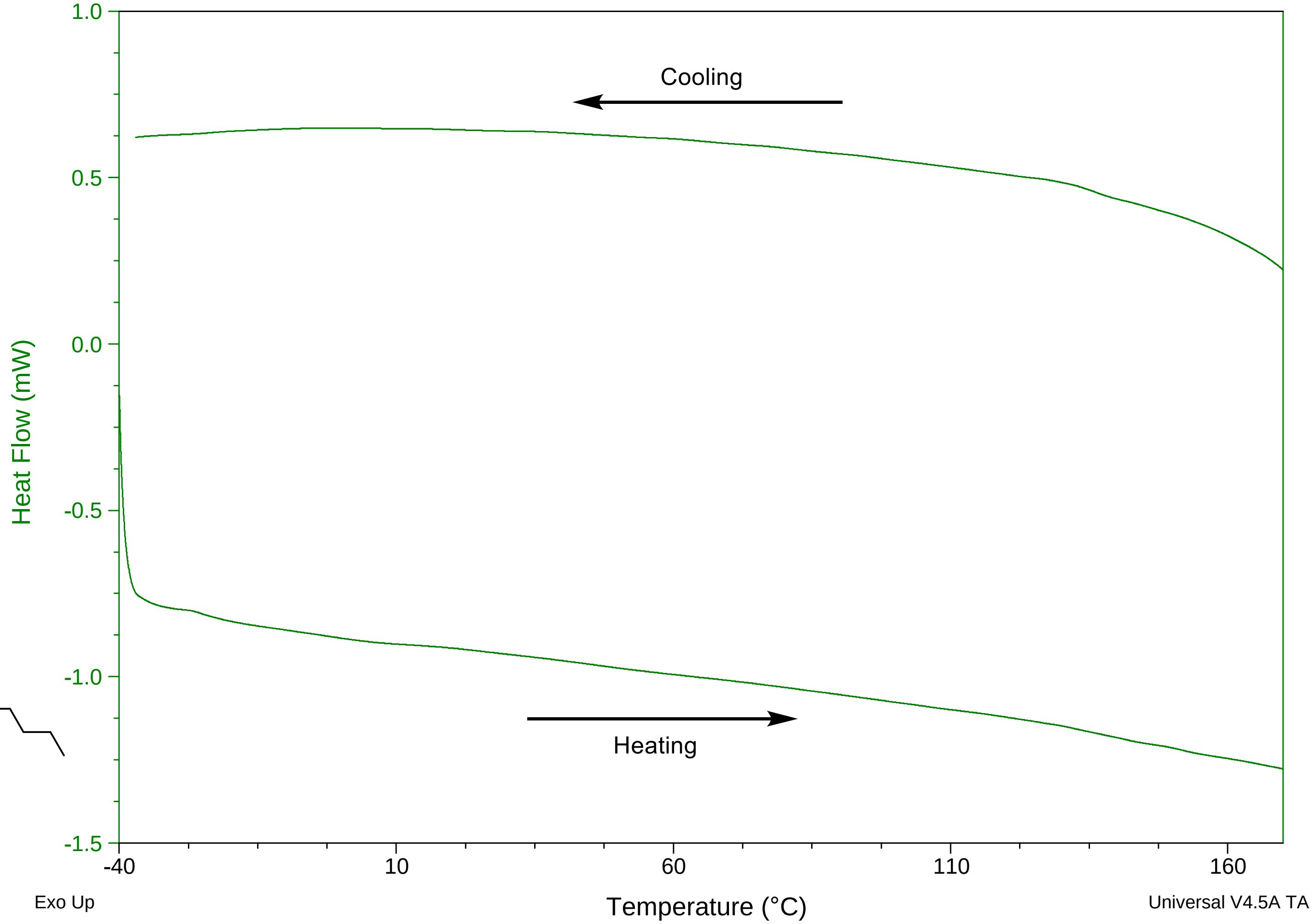

Figure S28. DSC Thermogram of **6**

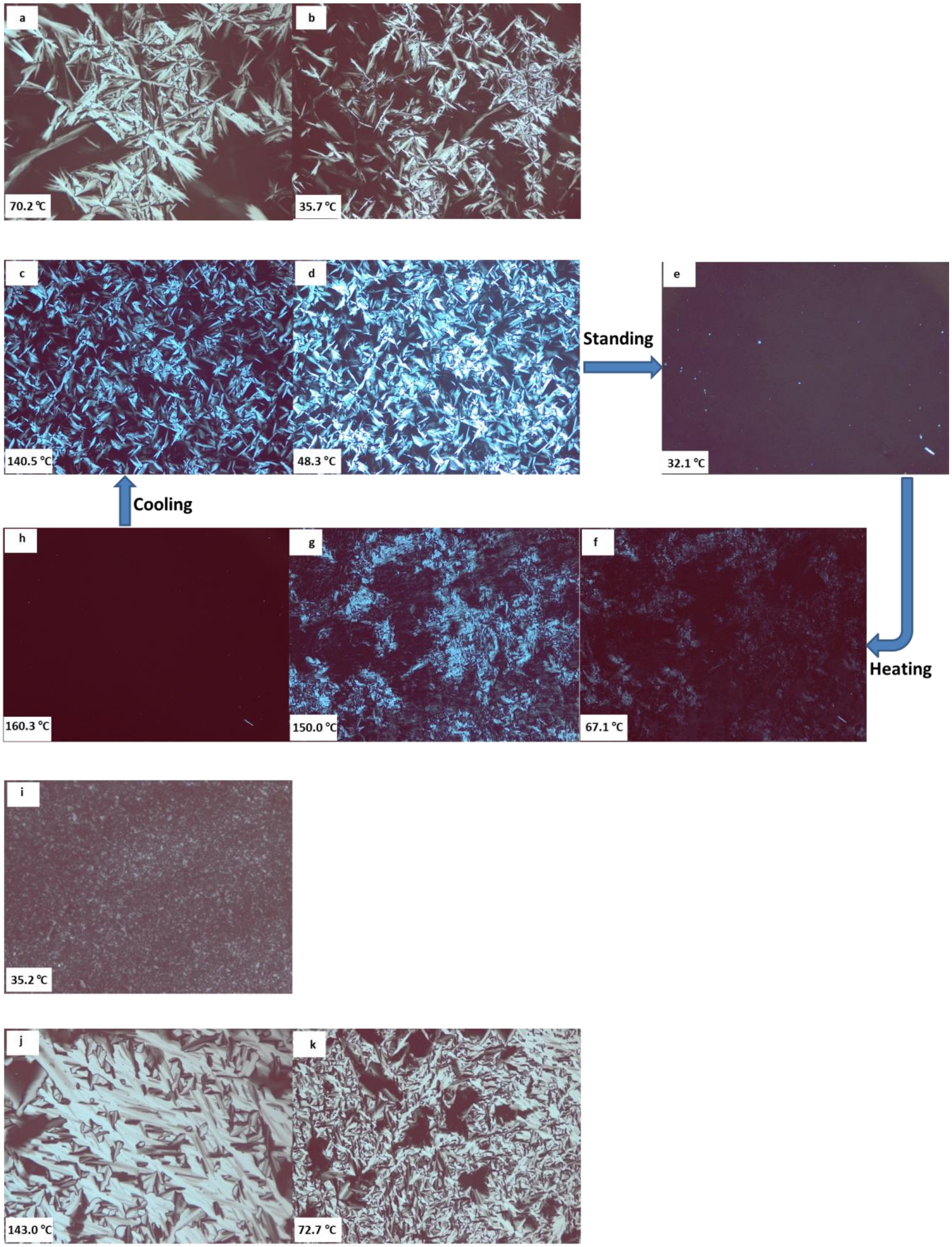

Figure S29. Cross-polarized micrographs of **3** during cooling cycle at 70.2 °C (**a**) and 35.7 °C (**b**), **4** during cooling cycle at 140.5 °C (**c**) and 48.3 °C (**d**), then standing at ambient temperature after several days (**e**), and reheating cycle to 67.1 °C (**f**), 150.0 °C (**g**) and finally at 160.3 °C (**h**), **5** during cooling cycle at 35.2 °C (**i**), **6** during cooling cycle at 143.0 °C (**j**) and 72.7 °C (**k**).

X-Ray Diffraction (XRD)

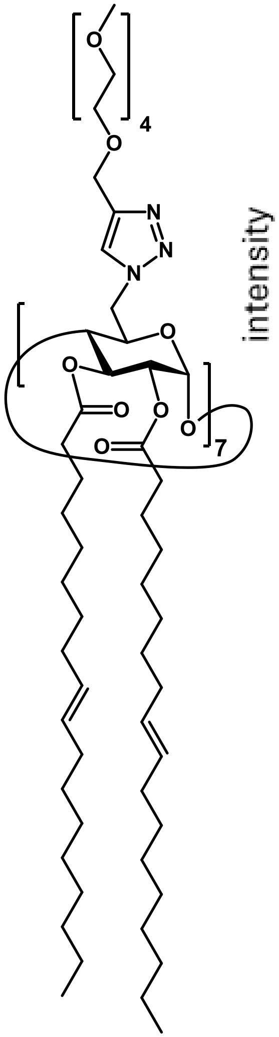

3

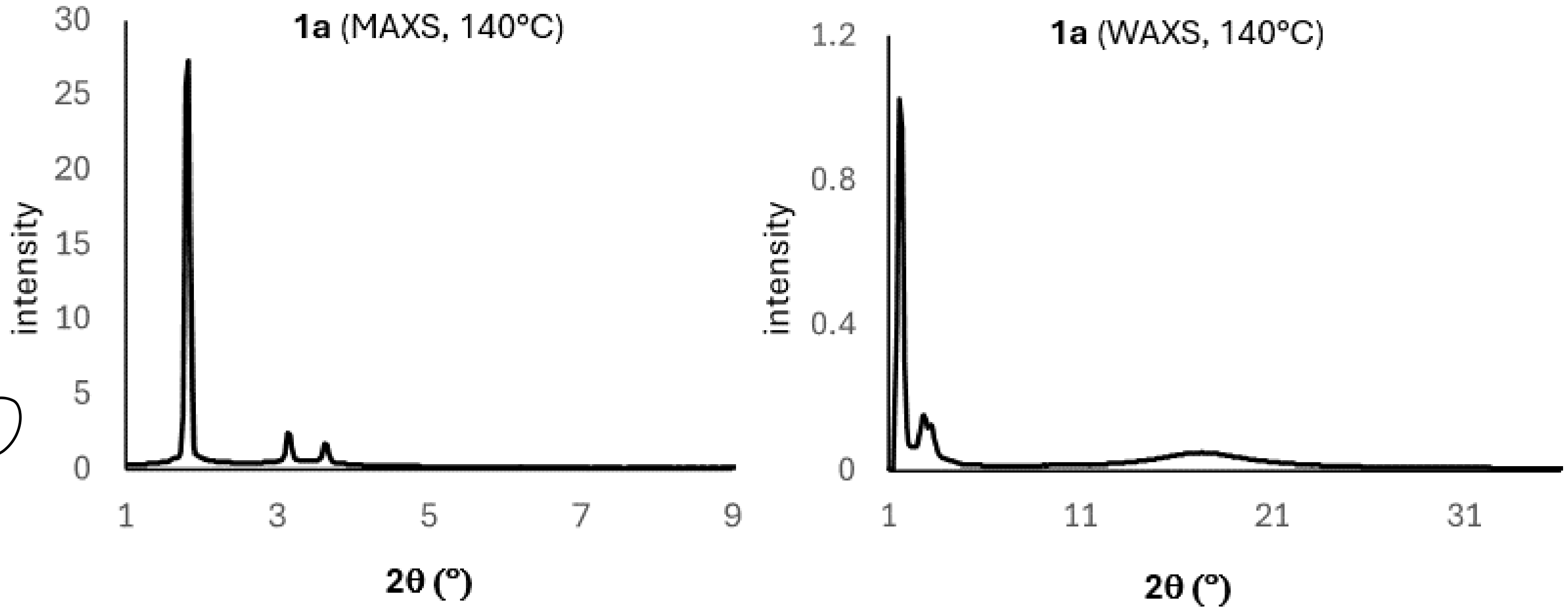

Figure S30. XRD of **3**

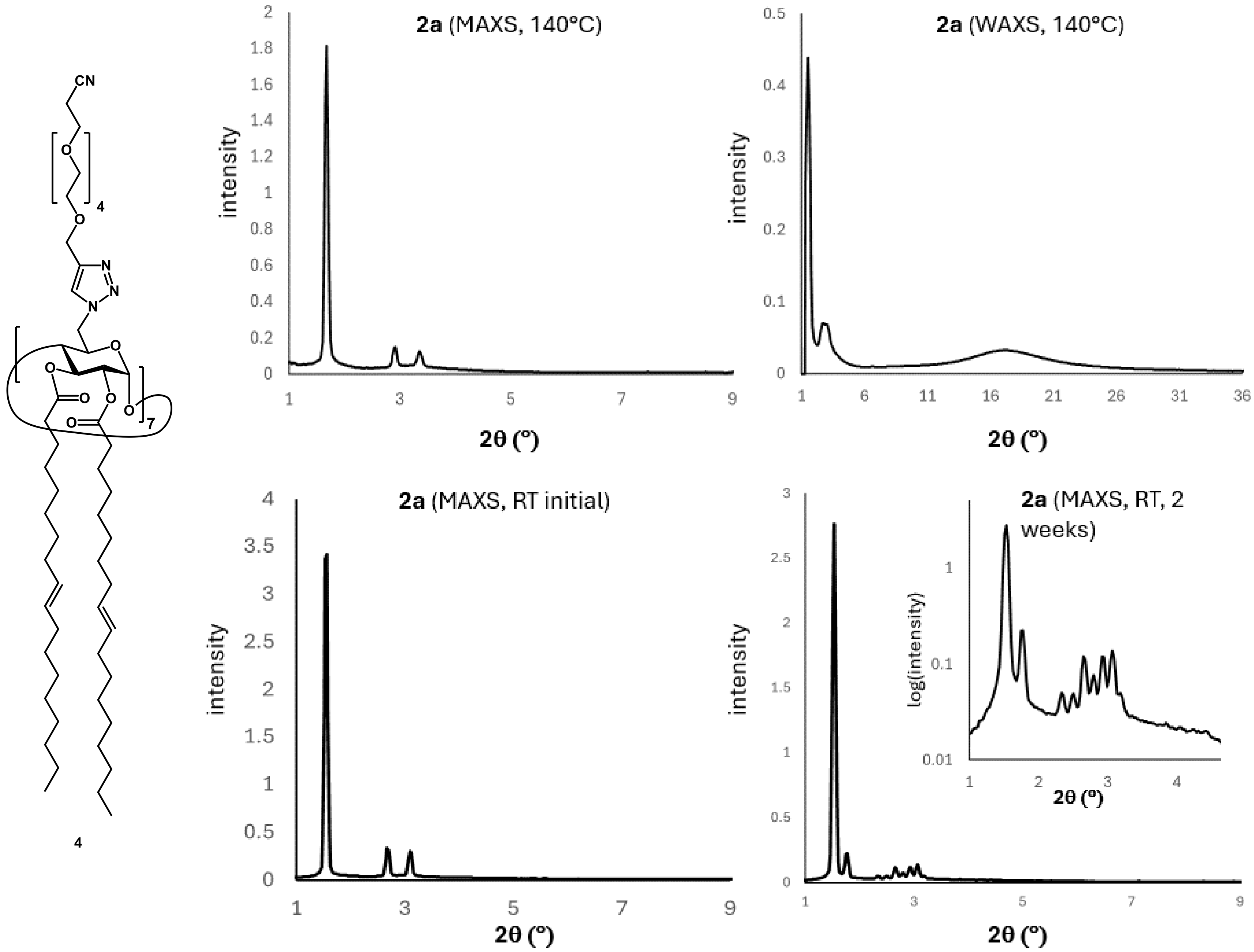

Figure S31. XRD of **4**

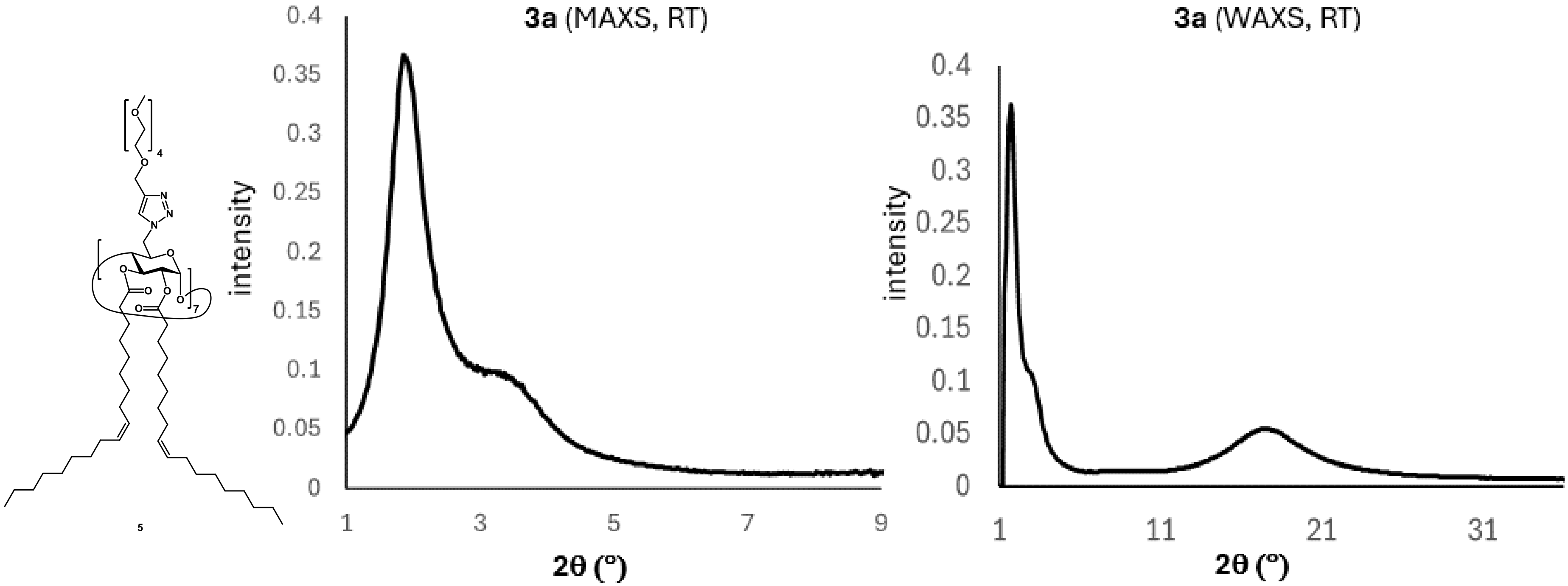

Figure S32. XRD of **5**

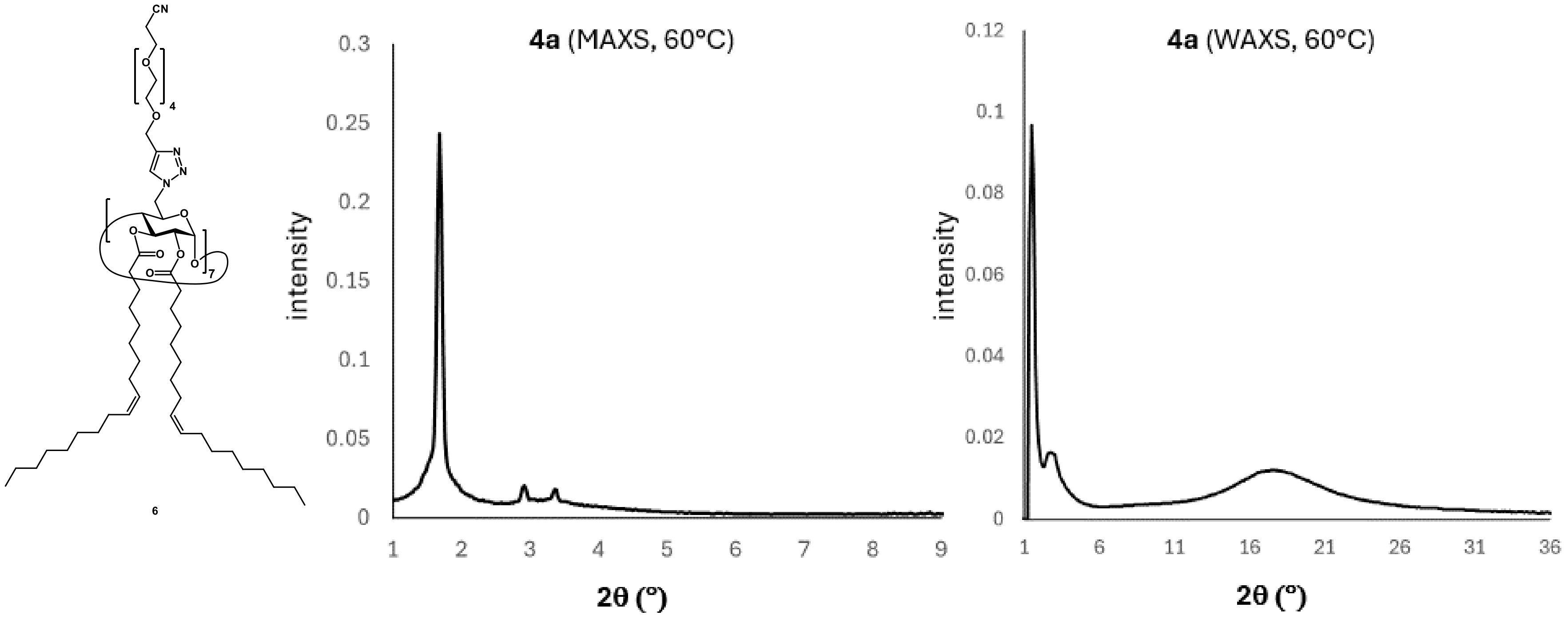

Figure S33. XRD of **6**
